# A phase I/II dose-escalation multi-center study to evaluate the safety of infusion of natural killer cells or memory T cells as adoptive therapy in coronavirus pneumonia and/or lymphopenia: RELEASE study protocol

**DOI:** 10.1186/s13063-021-05625-7

**Published:** 2021-10-02

**Authors:** I. García-García, P. Guerra-García, C. Ferreras, A. M. Borobia, A. J. Carcas, J. Queiruga-Parada, J. L. Vicario, I. Mirones, C. Solano, C. Eguizabal, B. Soria, A. Pérez-Martínez

**Affiliations:** 1 Clinical Pharmacology Department, University Hospital La Paz, Madrid, Spain; 2 Pediatric Hemato-oncology Department, University Hospital La Paz, Madrid, Spain; 3 Hospital La Paz Institute for Health Research, IdiPAZ, University Hospital La Paz, Madrid, Spain; 4 Faculty of Medicine, Universidad Autónoma de Madrid, Madrid, Spain; 5 Regional Blood Transfusion Centre, Madrid, Spain; 6 Hospital Clínico Universitario de Valencia/Instituto de Investigación Sanitaria INCLIVA, Universidad de Valencia, Valencia, Spain; 7 Research Unit, Basque Center for Blood Transfusion and Human Tissues, Osakidetza, Galdakao, Bizkaia Spain; 8 Cell Therapy, Stem Cells and Tissues Group, Biocruces Bizkaia Health Research Institute, Barakaldo, Bizkaia Spain; 9 Institute of Bioengineering, Miguel Hernández University, Elche, Alicante, Spain; 10 Health Research Institute-ISABIAL, Alicante University Hospital, Alicante, Spain; 11 University Pablo de Olavide, Sevilla, Spain

**Keywords:** COVID-19, Protocol, Allogeneic, Memory T cells, NK cells, Lymphopenia, Pneumonia, Safety

## Abstract

**Background:**

Moderate/severe cases of COVID-19 present a dysregulated immune system with T cell lymphopenia and a hyper-inflammatory state.

This is a study protocol of an open-label, multi-center, double-arm, randomized, dose-finding phase I/II clinical trial to evaluate the safety, tolerability, alloreactivity, and efficacy of the administration of allogeneic memory T cells and natural killer (NK) cells in COVID-19 patients with lymphopenia and/or pneumonia. The aim of the study is to determine the safety and the efficacy of the recommended phase 2 dose (RP2D) of this treatment for patients with moderate/severe COVID-19.

**Methods:**

In the phase I trial, 18 patients with COVID-19-related pneumonia and/or lymphopenia with no oxygen requirement or with an oxygen need of ≤ 2.5 liters per minute (lpm) in nasal cannula will be assigned to two arms, based on the biology of the donor and the patient. Treatment of arm A consists of the administration of escalating doses of memory T cells, plus standard of care (SoC). Treatment of arm B consists of the administration of escalating doses of NK cells, plus SoC.

In the phase II trial, a total of 182 patients with COVID-19-related pneumonia and/or lymphopenia requiring or not oxygen supplementation but without mechanical ventilation will be allocated to arm A or B, considering HLA typing. Within each arm, they will be randomized in a 1:1 ratio. In arm A, patients will receive SoC or RP2D for memory T cells plus the SoC. In arm B, patients will receive SoC or RP2D for NK cells plus the SoC.

**Discussion:**

We hypothesized that SARS-CoV-2-specific memory T-lymphocytes obtained from convalescent donors recovered from COVID-19 can be used as a passive cell immunotherapy to treat pneumonia and lymphopenia in moderate/severe patients. The lymphopenia induced by COVID-19 constitutes a therapeutic window that may facilitate donor engraftment and viral protection until recovery.

**Trial registration:**

ClinicalTrials.govNCT04578210. First Posted : October 8, 2020

**Supplementary Information:**

The online version contains supplementary material available at 10.1186/s13063-021-05625-7.

## Background

The infection caused by the 2019 novel severe acute respiratory syndrome coronavirus 2 (SARS-CoV-2) (formerly designated 2019-nCoV) was first discovered in Wuhan, China, in December 2019. On February 11, 2020, the World Health Organization (WHO) named the respiratory illness as 2019 coronavirus disease (COVID-19), and as of March 19, 2020, 234,073 cases of 2019-nCoV infection were confirmed worldwide, resulting in 9840 deaths [1]. The COVID-19 emergence has attracted global attention, being declared a public health emergency of international concern by the WHO [2, 3]. The effects of this disease range from asymptomatic infection to acute pneumonia, sometimes causing multi-organ failure leading to death [4, 5]. Although estimates of the fatality rate vary over time and are difficult to calculate, they are between 1 and 7%. Furthermore, long-term sequelae are still unknown, but to date, they include heart and lung tissue damage, heart failure, respiratory distress, anosmia, ageusia, pulmonary embolism, heart attack, stroke, cognitive impairment, anxiety, depression, post-traumatic stress disorder, sleep disturbance, joints and muscles pain, and fatigue, among others [3]. It has been reported that COVID-19 is more likely to occur in elderly patients with comorbidities and weaker immune systems [5], whereas the majority of children have had mild clinical manifestations and a favorable prognosis so far [6, 7].

Several drugs have been proposed as potential treatments for COVID-19. Some of these agents have been quickly tested in clinical studies and showed preliminary efficacy against the disease [8]. The Guidelines for the Prevention, Diagnosis, and Treatment of Novel Coronavirus-induced Pneumonia issued by the National Health Commission of the People’s Republic of China for tentative treatment of COVID-19 recommends antivirals including IFN-α, lopinavir/ritonavir, and ribavirin [9]. Chloroquine phosphate and arbidol are also included based on the preliminary outcomes of clinical studies [10]. However, most of these treatments have not proved to be effective enough to date [11]. Therefore, exploring effective therapies due to the worrisome situation is urgent. New drugs are currently undergoing clinical trials to assess if they can be used to treat COVID-19, such as favipiravir, remdesivir, and darunavir [8, 10]. Most of them are antiretroviral drugs and modulators of the inflammatory response. Nonetheless, few propose to use the potential of cell therapy for viral destruction and immune system recovery, which is a safe and cost-effective strategy successfully used in other diseases.

In this regard, several studies have shown increased amounts of pro-inflammatory cytokines in serum associated with pulmonary inflammation and extensive lung damage in COVID-19 [12, 13]. Furthermore, severe cases have higher leukocyte and neutrophil counts and neutrophil-to-lymphocyte ratio, in contrast with lower percentages of monocytes, eosinophils, basophils, lymphocytes, B cells, T cells, and NK cells [14–18]. The latter cells are a pivotal component of the innate immune system that can eliminate virally infected and malignant cells [19, 20]. Moreover, it has been demonstrated that NK cells play a role in another coronavirus infection—named SARS—originated in China in 2002. In SARS, both the number and function of NK cells correlated with the severity of the disease, meaning that coronavirus infection can alter the number and function of NK cells hampering their capacity to kill virally infected cells [21]. In addition, NK cells show characteristics that are typical of the adaptive immune system, as it has been proven by the existence of a pool of memory NK cells in mice and humans infected with cytomegalovirus (CMV) [22].

Furthermore, both helper T cells and suppressor T cells in patients with COVID-19 are below normal levels, being the reduction more prominent in severe cases [5, 13, 16, 23]. In particular, in severe cases, the percentage of naïve helper T cells increases, whereas the percentage of memory helper T cells decreases [24, 25]. These memory helper T cells can form a pool of long-lived memory cells, which upon re-encountering with their cognate antigen induce enhanced effector function resulting in increased protection of the host [26, 27].

Given the characteristics of NK cells and memory T lymphocytes, they can contribute to maintain an efficient antiviral immune response [19, 20, 28]. To accomplish this goal, two steps are needed: first, a rapid and well-coordinated innate immune response, and second, the differentiation of naïve helper T cells into effector and memory subsets [24, 25]. Recent and previous data have shown that infusions of donor memory T-lymphocytes (CD45RA^−^ T cells) are safe and constitute a simple measure to prevent infections in the setting of allogeneic hematopoietic stem cell transplantation (HSCT) [26, 29]. The infusion of a population of immune cells that have been in contact with COVID-19 and with immunological memory would create a quicker and more robust secondary immune response [26, 27, 30, 31].

Following this approach, this dose-escalation multi-center phase I/II clinical trial proposes the treatment of patients with bad prognosis due to SARS-CoV-2 infection, pneumonia, and/or lymphopenia, by using a cell therapy approach. It is an innovative and non-pharmacologic intervention, where NK cells or memory T lymphocytes will be infused from donors who have recovered from COVID-19 and have been free from disease for at least 14 days [32]. These procedures are routinely performed at the Haematology and Haemotherapy Services of many hospitals, meaning that there is extensive experience in this type of selective separation of cell populations [27]. For this reason, the cell therapy approach proposed in this study has shown to be essentially safe and can contribute to increase the therapeutic options available in the current SARS-CoV-2 emergency [26, 28, 29, 33]. It is a trial in accordance with usual clinical practice, being thus a low-level intervention trial.

A quick recovery of patients with pneumonia and/or lymphopenia is expected, based on the fact that the number and function of NK cells correlate with the severity of SARS, and this quick response is essential after a viral infection [19, 28]. Moreover, previous successful experiences treating other viruses such as CMV, Epstein-Barr virus (EBV), and human herpesvirus 6 (HHV-6) have been reported [34–36]. Regarding the pool of memory T cells, infusing them would increase its number in selected patients who have low levels of memory T cells due to the viral infection. These lymphocytes have long-life memory, which upon re-encountering with SARS-CoV-2 would induce an enhanced effector function resulting in greater protection of the patient [26, 27]. If this approach were successful, it would impact on saving thousands of lives, releasing hospital beds, reducing the costs of the National Health System, and improving the economy.

## Methods

The study protocol follows the SPIRIT recommendations.

### Aim, design, and setting

The study is a phase I/II multi-centric clinical trial. The main goal is to evaluate the safety and efficacy and to establish the recommended phase 2 dose of a new potential cell therapy for coronavirus-related pneumonia and/or lymphopenia that consists of a single infusion of NK cells or memory T cells from a healthy donor recovered from COVID-19.

Phase I comprises dose-escalating steps, whereas phase II aims to determine the efficacy of the treatment with a randomized, SoC-controlled setting. SoC was considered the best comparator in this study in order to demonstrate the efficacy of the intervention. Placebo injection was not considered due to technical difficulties in masking and the objective nature of the parameters defining recovery (temperature < 38°C armpit, and SpO2 > 94%, sustained for at least 24 h) or lymphopenia recovery through day 14.

The trial sponsor is Dr Antonio Pérez Martínez at University Hospital La Paz in Madrid. The study plans to include patients in seven centers in Spain (see Table 1), starting in April 2020 and with a planned study duration of 2 years. Follow-up is planned for 3 months.

**Table 1:** Study sites

Study site	Principal investigator
*Hospital Universitario La Paz*	Antonio Pérez Martínez
*Hospital Clinic de Barcelona*	Álex Soriano Viladomiu
*Hospital 12 de Octubre de Madrid*	María Liz Paciello Coronel
*Hospital Clínic de Valencia*	Carlos Solano Vercet
*Hospital General Universitario de Alicante*	Luis Manuel Hernández Blasco
*Hospital Universitario Cruces*	Juan José Mateos Mazón
*Hospital de Emergencias Enfermera Isabel Zendal*	Clara Hernández Blanco

Patients can enter the study if they meet the eligibility criteria, after signing the informed consent. Before initiating the trial, the investigator/institution must obtain approval/favorable opinion from the Independent Ethics Committee (EC) for the trial protocol, written informed consent form, consent form updates, subject recruitment procedures, and any other written information to be provided to subjects. Eligible subjects will be informed before the beginning of the study about the objectives and procedures, as well as the potential risks derived from the study participation.

The process of obtaining informed consent must be documented in the subject source documents.

**Fig. 1:**
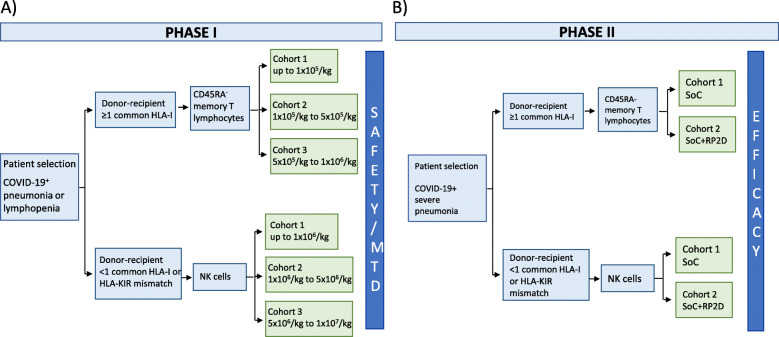
Trial flow chart of the two consecutive phases of the study

The study consists of two consecutive phases. In phase I (see Fig. 1A), 18 patients with pneumonia and/or lymphopenia related to COVID-19 will be assigned to two arms, based on the biological compatibility of the donor and the patient (if one or more HLA class I match between donor and recipient is present, patients will receive memory T cells—arm A; in case they have less than one HLA class I match or HLA-KIR mismatch, patients will receive NK cells—arm B). In both arms, participants are distributed in 3 dose-escalating cohorts. Arm A investigates the administration of a single dose of memory T cells plus the SoC, whereas arm B consists of the administration of a single dose of NK cells plus the SoC. To proceed to administer each subsequent dose, no signs of safety issues are required for at least 7 days after the cell infusion. In phase II (see Fig. 1B), a total of 182 patients with pneumonia and/or lymphopenia related to COVID-19 will be included. There will also be two arms based on the biology of the donor and the patient: arm A: allogeneic T memory cells and arm B: allogeneic NK cells based on the biological compatibility of the donor and the patient. Patients on each arm will be randomized (1:1) into two arms (SoC+RP2D) or to SoC. In arm A, patients will receive the SoC or the RP2D for memory T cells plus the SoC. In arm B, patients will receive the SoC or the RP2D for NK cells plus the SoC. The selected dose in each arm will be based on the phase I results. The development of the study protocol follows the Standard Protocol Items: Recommendations for Interventional Trials (SPIRIT) 2013 Checklist (Additional file 1). Figure 1 shows the trial flow chart, Table 2 the trial schedule for phase 1, and Table 3 the trial schedule for phase 2.

**Table 2:** Schedule of activities of phase I. Standard Protocol Items: Recommendations for Interventional Trials checklist (SPIRIT) figure

All the visits have a window of ± 1 day	Screening/D0	D1–D90 or until discharge	D7	D14	D21	D28	D90
**Informed consent**	x						
**SARS-CoV-2 PCR testing**			x	x	x	x	x
**Medical history**	x						
**Concomitant medications**	x	x	x	x	x	x	x
**Physical examination**	x	x	x	x	x	x	x
**Vital signs**	x	x	x	x	x	x	x
**Respiratory status**	x	x		x	x	x	x
**Hematology**	x	X^3^ (day 3)	x	x	x	x	
**Biochemistry**	x	X^3^ (day 3)	x	x	x	x	
**Markers of immunological function**^**1**^	x		x	x	x	x	x
**Blood sample for additional investigational studies**	x		x	x	x	x	
**Donor chimerism**		X^2^ (day 3)		X^2^	X^2^	X^2^	
**Pregnancy test**	x						
**Scales (NEWS and 7 point scale)**	x	x	x	x	x	x	x
Improvement according to the investigator/second dose**			x				
**DLTs**	x						
**Evaluation of patient recovery**				x			
**Adverse events**	X						

^1^Performed at screening and weekly

^2^Days 3 and 7. If donor chimerism persists by day 7, repeat on days 14, 21, and 28 and then continue weekly (until disappearance). Stop once it is negative

^3^Day 3

^**^Those patients showing clinical improvement at day 7 according to the investigator (based on respiratory status and blood-test result) who have not shown previous toxicities of grade 3 or higher *and* if donor chimerism does not persist can receive a second cycle with the same dose at day 7, if the investigator considers it appropriate

Patients will be followed until day 90, discharge, or death, whichever occurs earlier. However, if the patient is discharged before day 30, weekly ambulatory visits (days 7, 14, 21, and 28) will be performed if the investigator considers it appropriate

**Table 3:** Schedule of activities of phase II. Standard Protocol Items: Recommendations for Interventional Trials checklist (SPIRIT) figure

All the visits have a window of ± 1 day	Screening/D0	D1–D90 or until discharge	D7	D14	D21	D28	D90
**Informed consent**	x						
**SARS-CoV-2 PCR testing**			x	x	x	x	x
**Medical history**	x						
**Concomitant medications**	x	x	x	x	x	x	x
**Physical examination**	x	x	x	x	x	x	x
**Vital signs**	x	x	x	x	x	x	x
**Respiratory status**	x	x		x	x	x	x
**Hematology**	x	X^3^ (day 3)	x	x	x	x	
**Biochemistry**	x	X^3^ (day 3)	x	x	x	x	
**Markers of immunological function**^**1**^	x		x	x	x	x	x
**Blood sample for additional investigational studies**	x		x	x	x	x	
**Donor chimerism**		X^2^ (day 3)	x	X^2^	X^2^	X^2^	
**Pregnancy test**	x						
**Scales (NEWS and 7 point scale)**	x	x	x	x	x	x	x
**Evaluation of patient recovery**				x			
Improvement according to the investigator/second dose^**^			x				
**Adverse events**	x						

^1^Performed at screening and weekly

^2^Days 3 and 7. If donor chimerism persists by day 7, repeat on days 14, 21, and 28 and then continue weekly (until disappearance). Stop once it is negative

^3^Day 3

^**^Those patients showing clinical improvement at day 7 according to the investigator (based on respiratory status and blood-result tests) who have not shown previous toxicities of grade 3 or higher *and* if donor chimerism does not persist can receive a second cycle with the same dose at day 7, if the investigator considers it appropriate

Patients will be followed until day 90, discharge, or death, whichever occurs earlier. However, if the patient is discharged before day 30, weekly ambulatory visits (days 7, 14, 21, and 28) will be performed if the investigator considers it appropriate

### Study population

The target population for enrolment in the study will be patients with pneumonia and/or lymphopenia related to COVID-19. Participants are recruited from the contributor centers during the recruitment period.

A total of 182 patients will be included in the clinical trial. The objective is to enroll 18 patients in the dose-escalation stage (phase I), 9 patients per arm (3 patients in each cohort for both arms), and a total of 164 patients in phase II, 82 patients per arm. Additionally, the donors will be selected by the Regional Blood Transfusion Center during the first 5 months of the study.

Inclusion criteria:

- Male or female patients ≤ 80 years old
- Diagnosis of COVID-19 infection with laboratory confirmation by reverse-transcription PCR (RT-PCR) of SARS-CoV-2
- Onset of symptoms < 12 days prior to administration of study treatment
- *Phase I criteria*: Patients requiring hospitalization for COVID-19, with diagnosed *pneumonia* with chest radiograph or computed tomography imaging and/or *lymphopenia* (absolute lymphocyte counts below 1.2 × 10^9^cells /L) *AND* O_2_Sat ≤ 94% on room air at screening, no oxygen requirement or with an oxygen need of ≤ *2.5 lpm* in the nasal cannula
- *Phase II criteria*: Patients requiring hospitalization with *pneumonia* diagnosed with chest radiograph or computed tomography imaging or *lymphopenia* (absolute lymphocyte counts below 1.2 × 10^9^cells/L) *AND* O_2_Sat ≤ 94% on room air at screening, requiring or not oxygen supplementation (nasal cannula, oxygen mask with reservoir, non-invasive ventilation, etc.), but *excluding* mechanical ventilation
- Have a negative pregnancy test documented prior to enrolment (for females of childbearing potential)
- Be willing and able to comply with study procedures
- Patients must have the ability to comprehend and sign the informed consent
- Written informed consent obtained prior to any screening procedures

Exclusion criteria:

- Enrolled in another clinical trial for COVID-19
- Rapidly progressive disease with anticipated life expectancy <72 h
- Patients requiring mechanical ventilation
- Patients with multi-organ failure
- Moderate-severe (grade ≥ 3) organ impairment (liver, kidney), according to the criteria from the National Cancer Institute (NCI CTCAE version 5.0)
- Severe and/or uncontrolled concurrent medical disease that could cause unacceptable safety risks or compromise compliance with the protocol in the opinion of the clinical investigator
- Have a known history of human immunodeficiency virus infection, hepatitis B or hepatitis C; testing is not required in the absence of prior documentation or known history
- Pregnant or breastfeeding women, where pregnancy is defined as the state of a female after conception and until the termination of gestation, confirmed by a positive hCG laboratory test
- Any other condition that may interfere with the efficacy and/or safety evaluation of the trial according to the investigator’s opinion

The blood donor selection will be done by the Regional Blood Transfusion Center with the following criteria:

1. Male or female patients ≤ 65 years
2. SARS-CoV-2-positive patients by PCR during their disease
3. Complete resolution of symptoms at least 14 days prior to donation
4. Subjects must have at least one SARS-CoV-2-negative test by PCR from a nasopharyngeal swab, or, if available, a negative SARS-CoV-2 viremia tested by quantitative PCR in blood, before the blood donation
5. Donors must have HLA typing and KIR typing performed, in order to decide treatment assignment for patients
6. Subjects must be tested, at least, for the following: HBsAg, Anti-HBc, Anti-HCV, Anti-HIV 1 and 2, HCV, HBV**, HIV** (by NAT), CMV*, EBV*, Toxoplasma*, Syphilis (RPR or VDRL or FTA), and HTLV I/II**
  *At least IgG, ideally IgG and IgM
  **Mandatory for foreign patients, recommended for nationals

### Investigational product

The proposed treatment under investigation consists of the infusion of cells from donors in the recovery phase of the disease, who have been asymptomatic for at least 14 days prior to the donation, and with at least one SARS-CoV-2-negative test. Blood samples are purified, and cell subsets are obtained by magnetic immunoselection techniques. Blood donor apheresis will be split into 2 parts, memory T cells (CD45RA^−^) for arm A and NK cells (CD3^−^/CD56^+^) will be selected for arm B. Blood products from one donor can be utilized for more than one patient.

### Risks, adverse events (AEs)

The risk to which research subjects could be exposed in this trial can be minimized by adherence to the eligibility criteria and close clinical monitoring.

Potential risks associated with NK cells and/or memory T cell infusion may include, but are not limited to:

- Infusion reactions
- Side effects related to frozen products
- Graft-versus-host disease

Other unpredictable complications may occur, the most serious happening within the first month after T cell or NK cell infusion.

### Treatment assignment criteria and randomization

Donors and patients will be HLA tested and allocated in two different arms:

- Arm A: If one or more HLA class I match between donor and recipient are present, patients will receive memory T cells.
- Arm B: If the donor and recipient have less than 1 HLA class I match or HLA-KIR mismatch, they will receive NK cells.

For phase I, patients on each arm (A or B, according to the HLA compatibility) will subsequently undergo a correlative allocation into the different dose-escalating cohorts.

For phase II, eligible patients will be allocated to arm A or B considering HLA typing and within each arm they will then be randomized in a 1:1 ratio to the investigational arm (SoC+RP2D) or to SoC.

The randomization sequence was created by the Data Management Department, using SAS version 9.4 statistical software (procedure “PROC PLAN”) with a 1:1 allocation. The randomization seed was by default generated by the procedure from reading the time of day from the computer’s clock where the program was executed. Randomization will be done centrally through the electronic system RedCAP® in order to conceal the sequence until interventions are assigned. The randomization process is carried out based on the sequence generated by the SAS statistical program, with the assignment of treatment being blind. In this way, the program will only show “SoC (Standard of Care)” or “SoC (Standard of Care) + RP2D (Recommended Phase 2 Dose),” this assignment being blind. This is a simple randomization. As it is a multi-center study, the randomization is stratified by each site.

The patients who meet the selection criteria will be randomized by the members of the research team designated by each center to carry out the randomization, through the electronic system RedCAP®.

SoC medicines and supportive care are permitted according to institutional guidelines. All concomitant medications should be recorded, from the date the informed consent is signed until the patient is discharged.

### Treatment dosing and schedule

#### Single ascending dose cohorts (phase I)

All patients will receive the SoC according to institutional guidelines from each site for COVID-19. Additionally, they will receive the investigational treatment.

*Arm A*: If one or more HLA class I match between donor and recipient is present, patients will receive the corresponding number of memory T cells:

- Cohort 1: starting dose will be up to 1 × 10^5^/kg
- Cohort 2: 1 × 10^5^/kg to 5 × 10^5^/kg
- Cohort 3: 5 × 10^5^/kg to 1 × 10^6^/kg

*Arm B*: If donor and recipient have less than one HLA class I match or HLA-KIR mismatch, they will receive the following amount of NK cells:

- Cohort 1: starting dose will be up to 1 × 10^6^/kg
- Cohort 2: 1 × 10^6^/kg to 5 × 10^6^/kg
- Cohort 3: 5 × 10^6^/kg to 1 × 10^7^/kg

The decision to proceed to each subsequent dose level (next cohort) will be made based on safety and tolerability data from the prior lower dose level, in the moment in which the prior dosing group has completed the recruitment. Safety data for at least 7 days post-dose from all the subjects at the prior lower dose will be considered before dose escalation.

The 18 patients included in phase I will continue to be followed for possible DLTs during the 21 days following cell infusion.

#### Dose-level decisions for phase II

Dose-level decisions for phase II will be guided by safety and DLT data from phase I. Phase II will begin once RP2D is established based on the phase I data.

In both phases of the study, those patients showing clinical improvement at day 7 according to the investigator (based on respiratory status and blood-test results), who have not shown previous toxicities of grade 3 or higher and if donor chimerism does not persist, can receive a second cycle with the same dose at day 7, if the investigator considers it appropriate.

### Discontinuation criteria

If a subject discontinues the study, the investigator will try to perform all the needed evaluations to ensure that no adverse events occur.

- Study medication may be discontinued in the following instances:

- Discharge from the hospital/ institution
- Concomitant illness that would affect assessments of clinical status to a significant degree according to the investigator’s judgment
- Toxicity, or toxicity that, in the judgment of the investigator, compromises the ability to continue study-specific procedures or is considered not to be in the subject’s best interest
- Subject request to discontinue for any reason
- Subject’s non-compliance
- Pregnancy during the study

### Study outcomes

Phase I primary outcome is the occurrence of DLTs in all patients during the study treatment, until 21 days after cell infusion, and maximum tolerated dose (MTD). DLT is defined as any grade 3 or higher toxicity with an attribution of definitely or probably related to the infusion of the cells and any lower grade toxicity that increases to a grade 3 or higher as a direct result of the cell infusion.

Based on this, the *RP2D* will be defined as follows: The recommended dose will be the MTD unless no MTD is determined in the dose-escalation segment of the study. In the latter, the recommended dose will be the highest dose evaluated in the dose-escalation segment.

Co-primary outcome of phase I is the incidence and nature of DLT of a single infusion of NK or memory T cells from a healthy donor recovered from COVID-19 (dose escalation).

Phase II primary outcome is the incidence of patient recovery infusing adoptive NK cells or adoptive memory T cells. Recovery is defined as the proportion of participants in each group with normalization of fever and oxygen saturation (criteria for normalization: temperature < 38°C armpit, and SpO2 > 94%, sustained for at least 24 h) or lymphopenia recovery through day 14.

Secondary outcomes collected are additional biochemical information for the patient evolution (time to normal level of lymphocytes, time to negative SARS-CoV-2 test…) and general status of the patient (visits to intensive unit care, clinical status...). Exploratory objectives harbor those related with immune reconstitution (immunoglobulins, serum cytokines, T cells, NK cells, and B cells repertoire) and determination of donor chimerism by short tandem repeats and a variable number of tandem repeat markers using the ABI Prism 3130 System and immune lymphocyte reconstitution by multiparametric flow cytometry after adoptive therapy weekly during the first month after infusion.

Further information is in supplementary Table 2.

### Data collection and analysis plan

The handling, communication, and transfer of personal data of all the subjects participating in the study will be protected, complying with the basic ethical principles of Biomedical Research and with the applicable regulations: Regulation (EU) 2016/679 of the European Parliament and of the Council of 27 April 2016 on the protection of natural people with regard to the processing of personal data and on the free movement of such data, and repealing Directive 95/46/EC (General Data Protection Regulation) and LAW 41/2002, of 14 November, regulating patient autonomy and rights and obligations of information and clinical documentation.

An electronic case report form (CRF) has been designed using MACRO electronic Data Capture by Elsevier.

The statistical software R [R Core Team (2014). R: A language and environment for statistical computing. R Foundation for Statistical Computing, Vienna, Austria] will be used for statistical analysis.

Descriptive information will be presented as tabular summaries by the treatment group. Categorical data will be summarized by number and percentages, whereas continuous variables will be summarized by descriptive statistics. Fisher’s exact test will be used to determine statistical significance in the differences between groups. The results of the statistical analysis will be displayed in tables and as mean ± SD in figures. Significance levels will be expressed as *p*-values (*p < 0.05). The final trial data set will be available to statisticians and to all the main investigators.

Investigators will collect data from participants. A CRF will be completed for all patients that have given informed consent. All entries into the CRF are the responsibility of the investigator or a qualified designated staff member.

The Chief Investigator/Sponsor should retain the contents of the master file in paper or digital format for each clinical trial for at least 25 years after the end of the trial (according to current national legislation) or for a longer period if there are other applicable requirements.

Once the database has been locked, the Data Manager will produce an Adobe® Portable Document Format (PDF) of the MACRO® electronic Case Report Forms (eCRFs) to send to the sites and Sponsor.

The Data Manager performs a final inventory of all data management documentation and returns all documentation and study database within 30 days after the acceptance of the final analysis by the Chief Investigator/Sponsor. All study documents will be returned to the Sponsor with acknowledgment of receipt once the inventory is complete.

Once the study has been locked, all the information will be kept in data management files until the return of all documentation and no copy of it will be stored; therefore, whatever request related to the study will have to be done during this period.

### Sample size

A total of 182 patients will be included in this clinical trial. The plan is to enroll 18 patients in the first dose-escalation segment (phase I): 9 patients per arm (3 patients in each cohort, for both arms).

During phase I, three dose levels will be assessed. Dose level one will be up to 1 × 10^5^ memory T cells/kg in arm A and up to 1 × 10^6^ NK cells/kg in arm B. If all three recipients do not have any ≥ grade 3 toxicities associated with the experimental treatment within 7 days, the second cohort of 3 patients will receive 1–5 × 10^5^ memory T cells/kg in arm a and 1–5 × 10^6^ NK cells/kg in arm B. If none of these 3 patients has any ≥ grade 3 toxicities associated with the experimental treatment, then the third cohort of 3 patients will receive 0.5–1 × 10^6^ memory T cells/kg in arm A and 0.5–1 × 10^7^ NK cells/kg in arm B. If none of these 3 patients has any ≥ grade 3 toxicities associated with the experimental treatment, then the dose administered in cohort 3 will be the recommended dose for the phase II extension (RP2D). A total of 164 patients will be included in phase II: 82 patients per arm.

### Study procedures and assessment

The study procedures to be conducted for each subject enrolled in the study are presented in tabular form in Tables 2 and 3.

Subjects will receive a screening visit, within 2 days before randomization and dosing to determine eligibility for participation in the study. After the informed consent is signed, the assessments will be performed (SARS-CoV-2 testing, physical examination, respiratory status, blood samples, and recording all adverse events (AEs)). Study subjects who fulfill all the inclusion criteria and none of the exclusion criteria will be immediately randomized.

The screening visit will be followed by a baseline visit/infusion day visit (day 0), and further on, daily assessments from day 1 to 90 or until discharge will be performed as far as possible, considering the increased workload. Additional investigations (blood tests: hematology and biochemistry, markers of immunological function, and donor chimerism) and SARS-CoV-2 PCR will be performed weekly for 1 month.

Blood samples will be stored for research use. These samples will be processed to obtain the cells and a phenotypic study will be carried out to see the recovery in time of immunological markers using the flow cytometry technique. The functionality of infected COVID-19 cells and recovered cells will be studied by co-culture assays to see cytokine secretion by flow cytometry. In addition, plasma cytokines will be studied by protein assays.

### Protocol modifications and adherence

All protocol modifications must be documented in writing. Any protocol amendment will be identified by a consecutive number and must be approved and signed by the sponsor and the principal investigator.

If amendments are relevant, approval by the EC must occur before any changes can be implemented.

Amendments that are required for subject safety may be implemented immediately provided the health authorities are subsequently notified by protocol amendment and the reviewing EC is notified. The protocol amendment can be initiated either by the sponsor or by any principal investigator.

Investigators ascertain they will apply due diligence to avoid protocol deviations. If deviations occur, the investigator must inform the monitor and the consequences of such deviations will be reviewed and discussed among the team. All protocol deviations will be documented/recorded specifying reason, data, action taken, and consequences in patients and in the study. All documentation related to deviation will be stored in the investigator file.

### Data monitoring committee

The trial will be monitored by the personnel from the Spanish Clinical Research Network (SCReN). They will verify the correctness of the data collection and manage the data. An appropriate monitoring plan for this study has been developed. The monitor will visit the site to check the completeness of subject records, the accuracy of data capture/data entry, the adherence to the protocol and to Good Clinical Practice, and the progress of enrollment and to ensure that the study treatment is being stored, dispensed, and accounted for according to specifications. The investigator must give the monitor access to all relevant source documents for monitoring activities, audits, EC revisions, and inspections from Health Authorities.

The investigator must maintain source documents for each subject in the study, if applicable, consisting of case and visit notes (hospital or clinical medical records) containing demographic and medical information, laboratory data, and the results of any other tests or assessments. All information on CRFs must be traceable to these source documents in the subject’s file. The investigator must also keep the original informed consent form signed by the subject (a signed copy should be given to the subject).

### Data safety monitoring

The investigator will monitor and systematically collect the AEs starting from the date of signing the informed consent form until the final follow-up visit of each subject. The occurrence of AEs is sought by non-directive questioning of the subject at each visit during the study. Adverse events may also be detected through physical examination, laboratory test findings, or other assessments. There is also a Data Safety Monitoring Board (DSMB), who will periodically review and evaluate the accumulated study data for participant safety, study conduct and progress, and, when appropriate, efficacy, and will make recommendations concerning the continuation, modification, or termination of the trial.

The DSMB considers study-specific data as well as relevant background knowledge about the disease, test agent, or patient population under study.

### Dissemination of trial results

Anonymized individual participant data will be made available when the trial is complete, on request to the corresponding authors and approval by the scientific committee. After approval of a proposal, data will be shared through a secure online platform. The protocol will be registered in a publicly accessible database. In addition, after study completion and finalization of the study report, the results of this trial will be submitted for publication in a scientific journal or will be made public, complying with the Declaration of Helsinki.

## Discussion

Several drugs have been proposed as potential treatments for COVID-19. However, most of these treatments are not specific and have not proved to be effective enough. Currently, only steroids have been reported to decrease mortality in patients with severe COVID-19 [37]*.* Older patients and patients with pre-existing comorbidities are at higher risk for complications. Pneumonia and T cell lymphopenia are indicators of disease severity and higher death risk [38]*.* Here, we describe an innovative and non-pharmacologic intervention, where NK cells or memory T lymphocytes will be infused from donors who have recovered from COVID-19 and have been free from disease for at least 14 days. The aim of the study is to determine the safety and the efficacy of the RP2D of this treatment for patients with moderate/severe COVID-19.

The protective role of T cells has already been shown in previous SARS-CoV viruses [39]. Both helper T cells and suppressor T cells in patients with COVID-19 are below normal levels, being the reduction more prominent in severe cases. In severe cases, the percentage of naïve helper T cells increases, whereas the percentage of memory helper T cells decreases [40]. In this regard, several studies have shown increased amounts of pro-inflammatory cytokines in serum associated with pulmonary inflammation and extensive lung damage in COVID-19 [41]. In particular, severe cases have higher leukocyte and neutrophil counts and neutrophil-to-lymphocyte ratio, in contrast with lower percentages of monocytes, eosinophils, basophils, lymphocytes, B cells, T cells, and NK cells [42].

The lymphopenia induced by COVID-19 constitutes a therapeutic window that may facilitate donor engraftment and viral protection until recovery. We hypothesized that SARS-CoV-2-specific memory T-lymphocytes obtained from convalescent donors recovered from COVID-19 can be used as a passive cell immunotherapy to treat pneumonia and lymphopenia in moderate/severe patients [43]. These memory helper T cells can form a pool of long-lived memory cells, which upon re-encountering with their cognate antigen induce enhanced effector function resulting in increased protection of the host.

To date, no treatment with cell therapy has been approved for COVID-19; some are in experimental phases or have gone through pilot studies and include treatment with mesenchymal stem cells [44, 45] or NK cells. NK cells are lymphocytes of the innate immune system that can eliminate virally infected and malignant cells [19, 20]. Coronavirus infection can alter the number and function of NK cells hampering their capacity to kill virally infected cells. Clinical data has shown that COVID-19 disease severity is correlated with a reduction in the number and function of NK cells. The capacity of NK cells to recognize and eliminate viral infections such as CMV, hepatitis B virus (HBV), and hepatitis C virus (HCV) has been well described. In this regard, adoptive NK cell therapy for COVID-19 patients can improve survival and limit disease progression in moderate/severe cases.

## Trial status

The study protocol version 1.0 of the 1st of April 2020 was approved by the Ethics Committee CEIm Hospital Universitario La Paz (Identifier: Clinical Ethical Approval No. HULP-5579) on the 8th of April 2020. The current version is protocol version 5.0, 14 May 2021. Recruitment is ongoing. The first participant was recruited on the 4th of September 2020. The final participant is anticipated to be recruited in April 2022.

As of 23rd of February 2021, a total of 9 participants have received treatment in arm A of phase I (all of them at University Hospital La Paz). No DLTs were detected and no MTD was established; therefore, after data assessment by the Data Safety Monitoring Board (DSMB), the recommended dose for arm A of phase II has been established as 5 × 10^5^/kg to 1 × 10^6^/kg. As of 26 August 2021, a total of 27 participants have been included in arm A of phase II (14 have received SoC + RP2D for memory T cells and 13 have received the SoC). The enrolment is ongoing.

In future manuscripts derived from this protocol, author contributions will be determined according to who conceived and designed the study, who collected the data, who contributed to data analysis, and who wrote the paper. Medical writers might be used in the writing process.

## Supplementary Information

**Additional file 1.** RELEASE Protocol v5.0 14^th^ May 2021.

**Additional file 2.** Informed Consent v4.0 18^th^ March 2021.

**Additional file 3.** Supplementary Tables 1 and 2.

## Data Availability

No additional data or material is available.

## Abbreviations

SoC: Standard of care
RP2D: Recommended phase II dose
NK: Natural killer
DLT: Dose-limiting toxicity
CRF: Case report form
SARS-CoV-2: Severe acute respiratory syndrome coronavirus 2
WHO: World Health Organization
COVID-19: 2019 coronavirus disease
HSCT: Hematopoietic stem cell transplantation
CMV: Cytomegalovirus
EBV: Epstein-Barr virus
HHV-6: Human herpesvirus-6
RT-PCR: Reverse-transcription PCR
EC: Ethics Committee
AEs: Adverse events
SPIRIT: Standard Protocol Items: Recommendations for Interventional Trials
