# Supplementary material for: A phase I/II dose-escalation multi-center study to evaluate the safety of infusion of natural killer cells or memory T cells as adoptive therapy in coronavirus pneumonia and/or lymphopenia: RELEASE study protocol
Source: Trials. 2021 Oct 2;22:674. doi: 10.1186/s13063-021-05625-7 (PMC8487326; doi:10.1186/s13063-021-05625-7)
Supplement: Supplementary file 1 — Additional file 1. RELEASE Protocol v5.0 14th May 2021. [file 13063_2021_5625_MOESM1_ESM.docx]

**CLINICAL TRIAL PROTOCOL**

| **A phase I/II dose-escalation multi center study to evaluate the safety of infusion of NatuRal KillEr celLs or MEmory T cells as Adoptive therapy in coronaviruS pnEumonia and/or lymphopenia** |
| --- |
| **Protocol versión 5.0 14th May 2021** |

**Sponsor:** Dr. ANTONIO PÉREZ-MARTÍNEZ

**Protocol code:** RELEASE

**CONFIDENTIAL**

The information and data included in this protocol contains commercial secrets and privileged or confidential information that is the property of the Sponsor. No one is authorized to make this information public without written permission from the sponsor. These limitations apply to all information considered privileged or confidential that is facilitated to you in the future. This material may be divulged and used by the team and collaborators as needed to conduct the clinical trial.

**INDEX**

INDEX2

PROTOCOL SUMMARY5

Coordinating investigator5

Investigator team6

Clinical phase6

Independent Ethics Committee[6](#_Independent_Ethics_Committee)

Summary of trial rationale[6](#_Summary_of_trial)

Medical condition[8](#_Medical_condition)

Investigational product[8](#_Investigational_product)

Study design[8](#_Study_design)

Study treatment[9](#_Study_treatment)

Study population[9](#_Study_Population)

Sample size[9](#_Sample_size:)

Study duration[10](#_Study_Duration)

Study calendar[10](#_Study_Calendar)

Trial objectives[10](#_Trial_Objetives)

GENERAL INFORMATION11

Coordinating investigator11

Scientific committee[11](#_Scientific_Committee_1)

Investigator team[12](#_Investigator_team_1)

Data monitoring committee[12](#_Data_Monitoring_Committee)

Independent Ethics Committee[12](#_Independent_Ethics_Committee_1)

Study calendar[12](#_Study_Calendar_1)

BACKGROUND AND RATIONALE14

Introduction[14](#_Introduction)

New strategies: Cell Therapy [15](#_New_strategies:_Cell)

STUDY APPROACH18

Hypotheses[18](#_Hypotheses)

Study design[18](#_Study_design_1)

Trial objectives[22](#_Trial_Objectives)

Identified and potential risks to patients[22](#_Identified_and_Potential)

STUDY POPULATION24

Donor selection criteria[24](#_Donor_selection_criteria:)

Inclusion criteria[25](#_Inclusion_criteria)

Exclusion criteria[26](#_Exclusion_criteria)

INVESTIGATIONAL PRODUCT, DOSAGE AND MODE OF ADMINISTRATION27

Subject treatment assignment[30](#_Subject_Treatment_Assignment)

Prior and concomitant medication[30](#_Prior_and_concomitant)

Criteria for discontinuation of study treatment30

STUDY PROCEDURES AND ASSESSMENTS32

Screening visit[32](#_Screening_Visit)

Baseline / Day 1 Assessments[33](#_Baseline/Day_1_Assessments)

Daily study assessments[34](#_Daily_Study_Assessments)

Additional assessments[35](#_Additional_Assessments)

Schedule of activities phase I[36](#_Schedule_of_activities)

Schedule of activities phase II[37](#_Schedule_of_activities_1)

Follow up[38](#_Follow-up)

STUDY OUTCOME MEASURES39

Primary outcome[39](#_Primary_outcome:)

Secondary outcome[40](#_Secondary_outcomes_:)

Exploratory outcome[40](#_Exploratory_outcomes:)

SAFETY MONITORING AND REPORTING41

Definitions[41](#_DEFINITIONS)

Adverse event (AE)41

Adverse reaction (AR)41

Serious adverse event (SAE) and Serious Adverse Reaction (SAR)41

Classification of an adverse event[42](#_CLASSIFICATION_OF_AN)

Severity of an event42

Relationship to study intervention42

Expectedness42

Unexpected adverse reaction43

Suspected Unexpected Serious Adverse Reaction (SUSAR)43

Information on adverse events[43](#_INFORMATION_ON_ADVERSE)

SAE reporting[43](#_SAE_REPORTING)

SUSAR expedited reporting44

Expedited reporting of other relevant safety information[44](#_EXPEDITED_REPORTING_OF)

Pregnancy reporting[45](#_PREGNANCY_REPORTING)

ETHICAL CONSIDERATIONS45

General considerations[45](#_General_Considerations)

Informed consent[46](#_Informed_Consent)

Data management, confidentiality of trial documents and patients’ records[46](#_Data_management,_confidentiality)

Insurance[47](#_Insurance)

PRACTICAL CONSIDERATIONS48

Responsibilities of patients[48](#_Responsibilities_Of_Patients)

Responsibilities of the investigator[48](#_Responsibilities_Of_The)

Protocol adherence[48](#_Protocol_Adherence)

Monitorization, audit and inspection[48](#_Monitorization,_Audit_and)

Publication of the study protocol and results[49](#_Publication_Of_The)

Protocol amendments[49](#_Protocol_Amendments)

Ethics Committee (EC)[50](#_Ethics_Committee_)

DATA ANALYSIS AND STATISTICAL METHODS51

BIBLIOGRAPHY52

APPENDIX 1. The NEWS scoring system[55](#_APPENDIX_1._The)

APPENDIX 2. 7-point scale for clinical improvement[56](#_Appendix_2:_7-point)

APPENDIX 3. Declaration of Helsinki[57](#_APPENDIX_3:_Declaration)

**LIST OF FIGURES**

Figure 1 Expected timeline13

Figure 2 Study scheme21

Figure 3 Schematic representation of procedure for blood obtention25

Figure 4 Different types of HSCT performed in the past ten years (Pediatric Hemato-Oncology Department, University Hospital *La Paz*)27

**LIST OF TABLES**

Table 1 Number of NK cell and memory T cell infusions in the past two years (Pediatric Hemato-Oncology Department, University Hospital *La Paz*)28

Table 2 Schedule of activities Phase I36

Table 3 Schedule of activities Phase II37

1. **PROTOCOL SUMMARY**

**Sponsor:** Dr. ANTONIO PÉREZ-MARTÍNEZ*.*

**Protocol code:** RELEASE

## Study Title: A phase I/II dose-escalation multi-center study to evaluate the safety of the infusion of NatuRal KillEr celLs or MEmory T cells as Adoptive therapy in coronaviruS pnEumonia and/or lymphopenia (RELEASE)

**Coordinating Investigator**

Dr. Antonio Pérez Martínez

*Hospital Universitario La Paz.* Pediatric Hemato-Oncology Department

Paseo de la Castellana, 261 CP 28046 Madrid

Telephone 917277223/Fax 917277042

# email: [aperezmartinez@salud.madrid.org](mailto:aperezmartinez@salud.madrid.org)

# Scientific Committee

**Medical Director (Hospital *Universitario La Paz*)**

- Dr. Juan José Ríos

**Infectious Diseases Department**

- Dr. José Ramón Arribas
- Dr. Rocío Montejano

**Microbiology Department**

- Dr. Julio García Rodríguez

**Clinical Pharmacology Department (Hospital *Universitario La Paz*)**

- Dr. Antonio J. Carcas
- Dr. Alberto Borobia Pérez
- Dr. Irene García García
- Dr. Amelia Rodríguez Mariblanca
- Dr. Lucía Martínez de Soto

***Centro de Transfusión de la Comunidad de Madrid***

- Dr. José Luis Vicario
- Dr. Antonio Balas
- Dr. Félix García Sánchez

**Hematology Deparment (Hospital *Universitario La Paz*)**

- Dr. Raquel de Paz
- Dr. Mercedes Gassior

**Pediatric Hemato-Oncology Department (Hospital *Universitario La Paz*)**

- Dr. Pilar Guerra García
- Dr. Isabel Mirones Aguilar
- Dr. Antonio López
- Dr. Cristina Ferreras
- Dr. Adela Escudero
- Dr.Antonio Pérez Martínez

## Investigator team

| **Name** | **Department** | **Center** |
| --- | --- | --- |
| **Antonio Pérez Martínez** | Pediatric Hemato-Oncology Department | *Hospital Universitario La Paz* |
| **José Luis Vicario**  **Antonio Balas**  **Félix García Sánchez** |  | *Centro de Transfusión Comunidad de Madrid* |
| **Cristina Eguizábal**  **Lara Herrera**  **Silvia Santos**  **Mikel Pérez-Vaquero** | Cell Therapy, Stem cells and Tissues Research Unit, HLA laboratory and Production of Blood Components at CVTTH | *Centro Vasco de Transfusión y Tejidos Humanos (CVTTH)-IIS Biocruces Bizkaia.* |

## Study sites

See Appendix 4.

**Clinical phase:**

Phase I/II

## Independent Ethics Committee

This study will be reviewed by:

*CEIC Hospital Universitario La Paz*. Área Sanitaria 5 de la CCAA de Madrid

Paseo de la Castellana, 261, 28046 Madrid

e-mail: ceic.hulp.@salud.madrid.org

## Summary of trial rationale

There is an urgent need to evaluate, within clinical trials, the treatments currently used and with a potential efficacy for COVID-19.

This is a phase I/II clinical trial using adoptive cell therapy with NK cells or memory T cells in patients affected by COVID-19.

Severe cases with COVID-19 present a dysregulated immune system with T cell lymphopenia, specially NK cells and memory T cells, and a hyper-inflammatory state.

This clinical trial proposes the use of cell therapy for the treatment of patients with worse prognosis due to SARS-CoV-2 infection (those with pneumonia and/or lymphopenia). This is an innovative and a non-pharmacological intervention.

In this phase I/II trial, natural killer (NK) cells or memory T lymphocytes will be infused from donors who have recovered from COVID-19 and have complete resolution of symptoms for at least 14 days.

There will be two arms based on the biology of the donor and the patient:

1. Infusion of NK cells, which are cells of the innate immune system that can eliminate virally infected cells.
2. Infusion of memory T cells, which have the SARS-COV-2 memory T cell repertoire.

We expect a quick recovery of the patients with pneumonia or lymphopenia for two reasons:

1. NK cells act quickly after a viral infection. The number and function of NK cells correlates with the severity of another coronavirus infection, Severe Acute Respiratory Syndrome (SARS), originated in China in 2002.

Moreover, we have previous successful experience with other viruses such as CMV, EBV and HHV-6.

1. The pool of memory T cells will increase in patients. Memory T cell levels are low in these patients. These lymphocytes have long-life memory, which upon reencountering SARS-CoV-2 will induce enhanced effector function resulting in greater protection of the patient.

Patients who have recovered from COVID-19 are the ideal donor candidates because they have immune cells with memory against SARS-CoV-2. Therefore, the infusion of NK and memory T cells from these donors will increase the pool of cells with cytotoxicity to virally infected cells, and will increase the pool of memory cells that respond quicker to a previously encountered stimulus.

This will impact in saving thousands of lives, releasing hospital beds, reducing the costs of a national health system and improving the economy of a locked-down country.

Cell therapies are safe and cost-effective and successfully used in other diseases. We need new innovative treatments where others have failed.

##

## Medical condition

2019 novel coronavirus disease (COVID-19)

## Investigational product

Cell Therapy

## Study design

This Phase I/II **escalating-dose** clinical trial is a randomized study to determine **safety, tolerability, alloreactivity and efficacy** of cell therapy with adoptive cell therapy of NK cells or memory T cells in patients affected by COVID-19.

Donors will be patients who have recovered from COVID-19 and are currently disease -free.

There will be two arms based on the biology of the donor and the patient.

- **Arm A**: allogeneic T memory cells
- **Arm B:** allogeneic NK cells

There will be two consecutive phases in the study:

- The first dose escalation segment focuses on determining safety and the recommended dose for the second segment.

**Phase I:** Patients with SARS-CoV-2+ pneumonia and/or lymphopenia **and** O_2_Sat ≤ 94% on room air at screening, with no oxygen requirement or with an oxygen need of **≤ 2.5 lpm** in nasal cannula, will be selected.

This phase has a single ascending dose design with up to 3 planned dose levels for each arm.

- The second phase extends the safety study.

**Phase II:** Patients with SARS-CoV-2+ pneumonia and/or lymphopenia **and** O_2_Sat ≤ 94% on room air at screening, requiring or not oxygen supplementation (nasal cannula, oxygen mask with reservoir, non-invasive ventilation, etc), but **excluding** mechanical ventilation, will be selected.

The dose selected will be based on the phase I results.

Additionally to the investigational product, all patients will receive the local standard of care for COVID-19 treatment.

## Study treatment

**Phase I:**

- **Arm A**: patients will receive memory T cells:
- Cohort 1: Starting dose will be up to 1x10^5^/kg
- Cohort 2: 1x10^5^/kg to 5x10^5^/kg
- Cohort 3: 5x10^5^/kg to 1x10^6^/kg
- **Arm B**: patients will receive NK cells:
- Cohort 1: starting dose will be up to 1x10^6^/kg
- Cohort 2: 1x10^6^/kg to 5x10^6^/kg
- Cohort 3: 5x10^6^/kg to 1x10^7^/kg

The next cohort will open if all the patients in the previous cohort have been included and no safety issues have aroused during the follow-up after cell infusion (data of at least 7 days after cell infusion is required to proceed to each subsequent dose).

Those patients showing clinical improvement at day 7 according to the investigator (based on respiratory status and blood-test results), who haven’t shown previous toxicities of grade 3 or higher **and** if donor chimerism doesn´t persist, can receive a second cycle with the same dose at Day 7, if the investigator considers it appropriate.

**Phase II**:

- **Arm A.** Patients will be randomized to receive one of the following treatments:
- Standard of Care Treatment (SoC)
- SoC + RP2D (Recommended Phase 2 Dose) for memory T cells
- **Arm B.** Patients will be randomized to receive one of the following treatments:
- Standard of Care Treatment (SoC)
- SoC + RP2D for NK cells

Those patients showing clinical improvement at day 7 according to the investigator (based on respiratory status and blood-test results) who haven’t shown previous toxicities of grade 3 or higher, can receive a second cycle with the same dose at Day 7, if the investigator considers it appropriate.

## Study Population

The target population for enrolment in this study will be patients with pneumonia or lymphopenia related to COVID-19.

## Sample size:

A total of 182 patients will be included in this clinical trial.

The plan is to enroll 18 patients in the first dose escalation segment (phase I): 9 patients per arm (3 patients in each cohort, for both arms).

A total of 164 patients will be included in the phase II: 82 patients per arm.

## Study Duration

This study has been planned over a period of 12 months with an initial donor selection phase of 5 months.

Period of recruitment, cell therapy administration and follow-up of patients: from the first month until the end of the study.

The phase of results' evaluation and preparation of the documentation to be reported will take place during the last three months.

**End of study date**: The end of study date will be the date of the last visit of the last patient included.

## Study Calendar

- First patient inclusion: April 2020
- Last patient inclusion: April 2022
- Subject participation duration: 3 months
- Study duration: 2 years

## Trial Objetives

Primary objective:

- **Phase I**

To determine the RP2D of a single infusion of NK or memory T cells from a healthy donor recovered from COVID-19 (dose escalation).

- **Phase II**

To determine the efficacy of a single infusion of NK or memory T cells from a healthy donor recovered from COVID-19.

1. GENERAL INFORMATION

**Sponsor:** Dr. ANTONIO PÉREZ-MARTÍNEZ

**Protocol code:** RELEASE

**Coordinating Investigator**

Dr. Antonio Pérez Martínez

*Hospital Universitario La Paz.* Pediatric Hemato-Oncology Department

Paseo de la Castellana, 261 CP 28046 Madrid

Telephone 917277223/Fax 917277042

# email: [aperezmartinez@salud.madrid.org](mailto:aperezmartinez@salud.madrid.org)

# Scientific Committee

**Medical Director (Hospital *Universitario La Paz*)**

- Dr. Juan José Ríos

**Infectious Diseases Department**

- Dr. JR Arribas
- Dr. Rocío Montejano

**Microbiology Department**

- Dr. Julio García Rodríguez

**Clinical Pharmacology Department (Hospital *Universitario La Paz*)**

- Dr. Antonio J. Carcas
- Dr. Alberto Borobia Pérez
- Dr. Irene García García
- Dr. Amelia Rodríguez Mariblanca
- Dr. Lucía Martínez de Soto

***Centro de Transfusión de la Comunidad de Madrid***

- Dr. José Luis Vicario
- Dr. Antonio Balas
- Dr. Félix García Sánchez

**Hematology Deparment (Hospital *Universitario La Paz*)**

- Dr. Raquel de Paz
- Dr. Mercedes Gassior

**Pediatric Hemato-Oncology Department (Hospital *Universitario La Paz*)**

- Dr. Pilar Guerra García
- Dr. Isabel Mirones Aguilar
- Dr. Antonio López
- Dr. Cristina Ferreras
- Dr. Adela Escudero
- Dr. Antonio Pérez Martínez

## Investigator team

| **Name** | **Department** | **Center** |
| --- | --- | --- |
| **Antonio Pérez Martínez** | Pediatric Hemato-Oncology Department | *Hospital Universitario La Paz* |
| **Begoña Monge** | Tropical Medicine Department | *Hospital Ramón y Cajal* |
| **Juan Carlos García** | Hemato-Oncology Department | *Hospital Universitario Cruces* |
| **Tomas Carrascosa** | Hemato-Oncology Department | *Hospital Universitario de Galdakao* |
| **Carlos Solano** | Hematology Department | *Hospital Clinic de Valencia.* |
| **José Luis Vicario**  **Antonio Balas**  **Félix García Sánchez** |  | *Centro de transfusión Comunidad de Madrid* |
| **Cristina Eguizábal**  **Lara Herrera**  **Silvia Santos**  **Mikel Pérez-Vaquero** | Cell Therapy, Stem Cells and Tissues Research Unit, HLA laboratory and Production of Blood Components at CVTTH | *Centro Vasco de Transfusión y Tejidos Humanos (CVTTH)-IIS Biocruces Bizkaia.* |

## Study sites

## See Appendix 4

## Data Monitoring Committee

Personnel from the [Spanish Clinical Research Network](http://scholar.google.es/scholar?q=spanish+clinical+research+network&hl=es&as_sdt=0&as_vis=1&oi=scholart) (SCReN)

## Data Safety Monitoring Board

-Dra. Irene García García. Clinical Pharmacology Department University Hospital La Paz, Madrid, Spain.

-Dr. Martínez López. Hemato-oncology Department, University Hospital 12 de Octubre, Madrid, Spain.

-Dr. Alberto Borobia. Clinical Pharmacology Department University Hospital La Paz, Madrid, Spain.

## Independent Ethics Committee

*CEIC del Hospital Universitario La Paz*. Área Sanitaria 5 de la CCAA de Madrid

Paseo de la Castellana, 261, 28046 Madrid

e-mail: [ceic.hulp.@salud.madrid.org](mailto:ceic.hulp.@salud.madrid.org)

## Study Calendar

This study has been planned over a period of 12 months with an initial donor selection phase of 5 months.

Period of recruitment, cell therapy administration and follow-up of patients: from the first month until the end of the study.

Results' evaluation and preparation of the documentation to be reported will take place during the last three months.

See figure 1 for the timeline

- First patient inclusion: April 2020
- Last patient inclusion: April 2022
- Subject participation duration: 3 months
- Study duration: 2 years


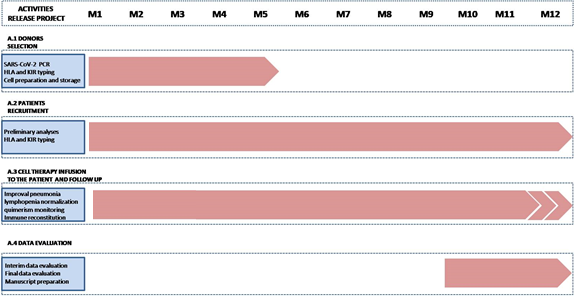
Figure 1: Expected timeline on the first 12 months.

1. BACKGROUND AND RATIONALE

## Introduction

The infection caused by the 2019 novel coronavirus SARS-CoV-2 (formerly designated 2019-nCoV) was first discovered in Wuhan, Hubei Province, China in December 2019. On 11 February 2020, the World Health Organization (WHO) named the illness as the 2019 coronavirus disease (COVID-19). By March 19, 234,073 cases with 2019-nCoV infection had been confirmed worldwide causing 9,840 deaths.

Emergence of 2019-nCoV has attracted global attention, and the WHO has declared the COVID-19 a public health emergency of international concern. The first case reported in Spain was in January 2020. Since then, and up to March 19, a total of 17,395 cases and 803 total deaths had been reported. The infection has suggested a case fatality rate of between 1-5%, although estimates of this are complicated.

Research thus far has revealed more than 30 agents including Western medicines, natural products, and traditional Chinese medicines that may have potential efficacy against COVID-19. Some of these agents have been quickly tested in clinical studies and demonstrated preliminary efficacy against COVID-19. Antivirals including interferon α (IFN-α), lopinavir/ritonavir, chloroquine phosphate, ribavirin, and arbidol have been included in the latest version of the Guidelines for the Prevention, Diagnosis, and Treatment of Novel Coronavirus-induced Pneumonia issued by the National Health Commission of the People’s Republic of China for tentative treatment of COVID-19.

The Guidelines have been revised five times since first being issued on January 15, 2020; the latest edition (the 6th edition) was issued on February 18, 2020. The fifth edition of the Guidelines recommends antivirals including IFN-α, lopinavir/ritonavir, and ribavirin for the treatment of COVID-19. Chloroquine phosphate and arbidol are included in the sixth edition of the Guidelines based on the preliminary outcomes of clinical studies. To date, most of these treatments have been useless. New drugs are currently undergoing clinical trials in treating COVID-19 as favipiravir, remdesivir and darunavir. A joint research team of the Shanghai Institute of *Materia Medica* and Shanghai Tech University performed drug screening in silicon and an enzyme activity test, and they reported 30 agents with potential antiviral activity against SARS-CoV-2 on January 25, 2020. These agents are indinavir, saquinavir, lopinavir, carfilzomib, ritonavir, remdesivir, atazanavir, darunavir, tipranavir, fosamprenavir, enzaplatovir, presatovir, abacavir, bortezomib, elvitegravir, maribavir, raltegravir, montelukast, deoxyrhapontin, polydatin, chalcone, disulfiram, carmofur, shikonin, ebselen, tideglusib, PX12, TDZD-8, cyclosporin A, and cinanserin. The same study also found that Chinese herbal medicines such as *Rhizoma Polygoni Cuspidati* and *Radix Sophorae Tonkinensis* might contain active ingredients against SARS-COV-2 causing COVID-19.

## New strategies: Cell Therapy

The current health emergency due to SARS-CoV-2 coronavirus infection requires the urgent development of therapeutic strategies to contain the pandemic.

Clinical trials currently underway are mostly based on the use of antiretroviral drugs and modulators of the inflammatory response, but few propose to use the potential of cell therapy for viral destruction and immune system recovery.

COVID-19 is more likely to occur in older patients with comorbidities, who have weaker immune functions. In fact, most of the children have mild clinical manifestations and a very good prognosis. Following this approach, several studies have shown that increased amounts of pro-inflammatory cytokines in serum were associated with pulmonary inflammation and extensive lung damage in COVID-19.

Severe cases had higher leukocyte and neutrophil counts, lower lymphocyte counts, higher neutrophil-to-lymphocyte ratio as well as lower percentages of monocytes, eosinophils, and basophils.

Lymphocytes, total number of B cells, T cells and Natural Killer (NK) cells are significantly decreased in patients with COVID-19, being more evident in severe cases. NK cells are lymphocytes of the innate immune system that can eliminate virally infected and malignant cells.

It has been demonstrated that NK cells played a role in another coronavirus infection - SARS- originated in China in 2002. In SARS, both the number and function of NK cells correlated with the severity of SARS, meaning that coronavirus infection can alter the number and function of NK cells hampering their killing potential to virally infected cells (National Research Project for SARS, Beijing Group). In addition, NK cells play a key role in viral control of HHV-6 and HIV+ resident cells.

NK cells also show characteristics that are typical of the cells of the adaptive immune system. In mouse and humans, the existence of a pool of memory NK cells, when infected with cytomegalovirus, has been proven.

Furthermore, both helper T cells and suppressor T cells in patients with COVID-19 were below normal levels, being more relevant in severe cases. Moreover, the percentage of naïve helper T cells increased and memory helper T cells decreased in severe cases. Memory T cells can form a pool of long-lived memory cells, which upon reencountering with their cognate antigen, induce enhanced effector function resulting in greater protection of the host.

Recent and previous data from our group and others have shown that infusions of donor memory T-lymphocytes, CD45RA- T cells, are safe and constitute a simple measure to prevent infections in the setting of allogeneic hematopoietic stem cell transplantation (HSCT). This immunological memory will create a more rapid and robust secondary immune response to COVID-19.

In summary, to maintain an efficient antiviral immune response, we need two mechanisms: first, we need both a rapid and well-coordinated innate immune response since it is the first line of defense against viral infections; and second, we need the differentiation of naïve CD4+ T-cells into effector and memory subsets. At the same time, the infusion of a population of immune cells that has been in contact with COVID-19 and with immunological memory will create a quicker and more robust response. This way, we can give hope to patients suffering from COVID-19 with bad prognosis.

This clinical trial proposes the treatment of patients who have a worse prognosis due to SARS-CoV-2 infection, pneumonia and/or lymphopenia, by using a cell therapy approach.

This is an innovative and a non-pharmacologic intervention.

In this phase I/II trial, natural killer (NK) cells or memory T lymphocytes will be infused from donors who have recovered from COVID-19 and are disease free for at least 14 days.

The technical advantage is that these procedures are routinely performed at the Hematology and Hemotherapy Services of many hospitals. There is extensive experience in this type of selective separation of cell populations due to the remarkable hematopoietic stem cell transplantation activity. For this reason, the cell therapy strategies proposed in this study have shown to be essentially safe and can contribute to increasing the therapeutic options available in the current emergency situation due to the SARS-CoV-2 infection.

We expect a quick recovery of the patients with pneumonia or lymphopenia for two reasons:

1. NK cells are cells that act quickly after a viral infection. The number and function of NK cells correlated with the severity of another coronavirus infection (SARS) originated in China in 2002. Besides, we have previous successful experience with other virus such as CMV, EBV and HHV-6.
2. The pool of memory T cells will increase in the patients. Memory T cell levels are low in these patients. These lymphocytes have long-life memory, which upon reencountering SARS-CoV-2 will induce enhanced effector function resulting in greater protection of the patient.

This will impact in saving thousands of lives, releasing hospital beds, reducing the costs of a national health system and improving the economy of a lockdown country.

Scientific Impact: This virus has presented as a new challenge for researchers and society.

Cell therapies are safe and cost-effective and successfully used in other diseases.

We need new innovative treatments where others have failed.

1. STUDY APPROACH

## Hypotheses

Our hypothesis is that patients who have recovered from COVID-19 should be the ideal donor candidates to restore the dysregulated immune system in patients with positive SARS-CoV-2. This will be achieved by the infusion of NK cells and/or memory T cells containing donor pathogen-specific memory NK and T-cell repertoire, including the SARS-CoV-2 repertoire.

Severe cases and adverse outcomes in COVID-19 present a dysregulated immune system with T cell lymphopenia, specially NK cells and memory T cells, and a hyper-inflammatory state. Therefore, the infusion of NK and memory T cells will increase the pool of cells with cytotoxicity to virally infected cells, and will increase the pool of memory cells that respond quicker to a previously encountered stimulus.

**Primary Hypothesis:**

- Adoptive cell therapy of NK cells or memory T cells will be safe and beneficial in COVID-19 patients.

**Secondary Hypothesis:**

- There will be an improvement of pneumonia symptoms in the next 2 weeks following infusion.
- There will be a trend to normalize lymphopenia, and immune dysregulation.

## Study design

## Open label, multicenter, double-arm interventional randomized phase I/II clinical trial study.

This Phase I/II escalating-dose clinical trial is a randomized study to determine RP2D, safety, tolerability, alloreactivity and efficacy of cell therapy with allogeneic NK cells (arm A) and T memory cells (arm B) from blood donors who have recovered from COVID-19 and are currently disease-free.

Donors and patients will be HLA tested and allocated into two different arms:

- **Arm A**: If one or more HLA class I match between donor and recipient are present, patients will receive memory T cells.
- **Arm B**: If donor and recipient have less than 1 HLA class I match or HLA-KIR mismatch, they will receive NK cells.

There will be two consecutive phases in the study:

1. The first dose escalation segment focuses on determining safety and the recommended dose for the second segment.
2. Phase II focuses on efficacy and safety.

**Phase I:**

Patients with SARS-CoV-2+ pneumonia or lymphopenia **and** O_2_Sat ≤ 94% on room air at screening, with no oxygen requirement or with an oxygen need of **≤ 2.5 lpm** in nasal cannula, will be selected.

This phase has a single ascending dose design with up to 3 planned dose levels:

- **Arm A:** patients will receive memory T cells:
  - Cohort 1: Starting dose will be up to 1x10^5^/kg
  - Cohort 2: 1x10^5^/kg to 5x10^5^/kg
  - Cohort 3: 5x10^5^/kg to 1x10^6^/kg
- **Arm B:** patients will receive NK cells:
  - Cohort 1: starting dose will be up to 1x10^6^/kg
  - Cohort 2: 1x10^6^/kg to 5x10^6^/kg
  - Cohort 3: 5x10^6^/kg to 1x10^7^/kg

Additionally to the investigational product, all patients will receive the local standard of care for COVID-19 treatment.

The next cohort will open if all the patients in the previous cohort have been included and no safety issues have aroused during the follow-up after cell infusion (data of at least 7 days after cell infusion is required to proceed to each subsequent dose).

Those patients showing clinical improvement at day 7 according to the investigator (based on respiratory status and blood-test results) who haven’t shown previous toxicities of grade 3 or higher, can receive a second cycle with the same dose at Day 7, if the investigator considers it appropriate.

The 18 patients in the Phase I will continue to be followed for possible DLTs during the 21 days following cell infusion.

**Phase II:**

Patients with SARS-CoV-2+ pneumonia or lymphopenia **and** O_2_Sat ≤ 94% on room air at screening, requiring or not oxygen supplementation (nasal cannula, oxygen mask with reservoir, non-invasive ventilation, etc) but **excluding** mechanical ventilation, will be selected.

- **Arm A**. Patients will be randomized to receive one of the following treatments:
  - Standard of Care Treatment (SoC)
  - SoC + RP2D for memory T cells
- **Arm B.** Patients will be randomized to receive one of the following treatments:
  - Standard of Care Treatment (SoC)
  - SoC + RP2D for NK cells

Phase II will begin once RP2D is established based on Phase I data.

Those patients showing clinical improvement at day 7 according to the investigator (based on respiratory status and blood-test results) who haven’t shown previous toxicities of grade 3 or higher, can receive a second cycle with the same dose at Day 7, if the investigator considers it appropriate.

See **Figure 2** for a schematic representation of the infusion regimen at each stage.

Figure2: Study scheme

## Trial Objectives

**Primary objective:**

**Phase I**

- To determine the RP2D (recommended Phase 2 dose) of a single infusion of NK or memory T cells from a healthy donor recovered from COVID-19 (dose escalation).

**Phase II**

- To determine the efficacy of a single infusion of NK or memory T cells from a healthy donor recovered from COVID-19.

**Secondary objectives:**

- To determine the dose-limiting toxicity (DLT) of a single infusion of NK or memory T cells from a healthy donor recovered from COVID-19.
- To determine the safety and tolerability of a single infusion of NK or memory T cells from a healthy donor recovered from COVID-19.
- To assess the efficacy of NK cells and memory T cell to normalize lymphopenia.
- To assess the time to negativity of SARS-CoV-2 polymerase chain reaction (PCR) with adoptive NK cells.
- To assess the time to negativity of SARS-CoV-2 PCR with adoptive memory T cells.

**Exploratory objectives:**

- **Co-primary objective**: to monitor donor chimerism and immune lymphocyte reconstitution after adoptive therapy during the 3 months following the infusion.
- Immunological profiling of patients responding to therapy.

## Identified and Potential Risks to Recipients

The risk to subjects in this trial may be minimized by adherence to the eligibility criteria and close clinical monitoring.

Potential risks associated with NK cells and/or memory T cell infusion include, but are not limited to:

## Infusion reactions:

## The side effects of infusion directly related to NK cells or memory T cell infusion are rare and short-lived but may include: cytokine release syndrome similar to a flu-like syndrome (headache, fever, chills, severe nausea/ vomiting, diarrhea, muscle and joint pain) or worsening of respiratory distress.

## Side effects related to frozen product:

## If the cell product is frozen, there are some side effects of infusion commonly related to the small amount of cryoprotectant present: flushing, itch, headache, fever, nausea and vomiting, abdominal cramps, diarrhea, high or low blood pressure, changes in the heart rate, chest discomfort, cough, shortness of breath.

In the case of cryopreserved cell infusions, premedication will be administered 30-60 minutes before cell infusion in order to prevent infusion reactions related to cryoprotectants, such as dimethyl sulfoxide.

## Graft-versus-host disease

## Graft-versus-host disease (GVHD) is a condition where the immune system of the donor attacks the recipient’s tissues and may range from mild to very severe. It can involve skin, liver and gastrointestinal tract in the acute phase and may become chronic causing dry mouth, dry eyes or skin changes. Considering the characteristics of the investigational products in this protocol we expect the incidence of clinically significant GVHD (grade III to IV) to be very low (<1%).

## Other complications that are unforeseeable may also occur. The most serious complications usually occur within the first month after T cell or NK cell infusion.

1. STUDY POPULATION

The target population for enrolment in the study will be patients with pneumonia or lymphopenia related to COVID-19.

- Pneumonia will be diagnosed as an acute respiratory disorder characterized by the presence of cough and at least one of the following: new-onset focal chest signs, fever for more than 4 days or dyspnea/tachypnea.
- Lymphopenia will be defined as lymphocyte counts below 1.2×10⁹.

A total of 182 patients will be included in this clinical trial. The plan is to enroll 18 patients in the first dose escalation segment (phase I): 9 patients per arm (3 patients in each cohort for both arms).

A total of 164 patients will be included in the phase II: 82 patients per arm.

**Sample Size**

Sample size has been calculated under the hypothesis of superiority of intervention versus standard of care assuming a one tailed alfa error of 0.05 and beta error of 0.2. According to the study with Remdesivir by Beigel et al (PMID: 32445440; DOI: 10.1056/NEJMoa2007764), a 42% of patients with basal punctuation of 4 to 6 at day 15 in the WHO ordinal scale recovered. A similar estimation (40%) is made by Rai et al (Rai, S.N., Qian, C., Pan, J. et al. Statistical design of Phase II/III clinical trials for testing therapeutic interventions in COVID-19 patients. BMC Med Res Methodol 20, 220 (2020). https://doi.org/10.1186/s12874-020-01101-z) for patients on intermediate risk (3 to 5 in this same scale). Based on these assumptions the table below shows the sample size for relative risks of 0.7 and 0.65 and recovery percentage of 40%, 45% and 50% in the standard of care group. Therefore, the objective is to recruit 82 patients per arm.

| **RR** | **P1** | **P2** | **SEnvelop** |
| --- | --- | --- | --- |
| 0.7 | 40 | 57.14 | 104 |
| 0.7 | 45 | 64.29 | 82 |
| 0.7 | 50 | 71.43 | 64 |
| 0.65 | 40 | 61.54 | 64 |
| 0.65 | 45 | 69.23 | 50 |
| 0.65 | 50 | 76.92 | 37 |

P1: percentage of recovery in standard of care group;

P2: percentage of recovery in intervention group.

**Statistical analysis.**

The crude probability of recovery at 14^th^ day will be compared by Chi-Square test. A covariate‐adjusted estimation of the ratio of probability of recovery in experimental treatment to standard of care will be based on the standardization approach of Ge et al. (2011; Ge, M., Durham, L.K., Meyer, R.D., Xie, W. and Thomas, N. (2011) Covariate‐adjusted difference in proportions from clinical trials using logistic regression and weighted risk differences. Drug Information Journal, 45, 481–493). Covariates to be included will be age, sex, basal punctuation in WHO scale at baseline and presence of cardiovascular risk factors.

## Donor selection criteria:

The blood donor selection will be done by the Regional Blood Transfusion Center with the following criteria:

1. Male or female patients ≤ 65 years
2. Subjects SARS-CoV-2+ tested by PCR+ during their disease
3. Complete resolution of symptoms at least 14 days prior to donation
4. Subjects must have at least one SARS-CoV-2 negative test by PCR from a nasopharyngeal swab, or, if available, a negative SARS-CoV-2 viremia tested by quantitative PCR in blood, before the blood donation.
5. Donors must have HLA typing and KIR typing performed, in order to decide treatment assignment for patients.
6. Subjects must be tested, at least, for the following:


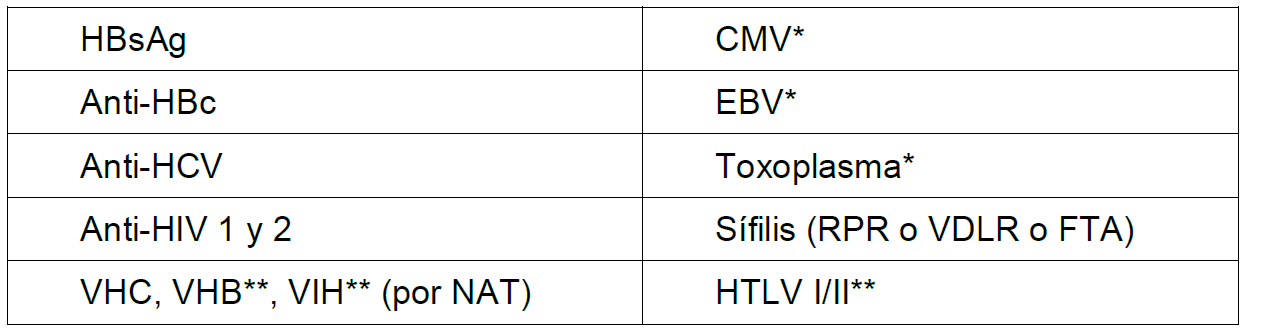


* At least IgG, ideally IgG and IgM

** Mandatory for foreign patients, recommended for nationals

Blood donor apheresis will be split into 2 parts, NK cells (CD3-/CD56+) will be selected for Arm 1 and memory T cells (CD45RA-) for Arm 2.

Both blood products will be infused freshly whenever possible. If necessary, the product will be cryopreserved and frozen for future use.

Blood product from one donor can be utilized for more than one patient.

Donors and patients will be HLA tested. If one or more HLA class I match between donor and recipient is present, they will receive memory T cells. If donor and recipient have less than 1 HLA class I match or HLA-KIR mismatch, they will receive NK cells.

See Figure 3.

******Figure 1. Schematic representation of procedure for blood donor obtention**

## Inclusion criteria

Patients eligible for inclusion in this study have to meet all of the following criteria:

- Male or female patients ≤ 80 years of age.
- Patient with diagnosis of COVID-19 infection with laboratory confirmation by reverse-transcription PCR (RT-PCR) of SARS-CoV-2 or other methods used according to each center protocol prior to study entry.
- Onset of symptoms < 12 days prior to administration of study treatment.
- **Phase I criteria:** Patients requiring hospitalization for COVID-19, with **pneumonia** diagnosed with chest radiograph or computed tomography imaging or **lymphopenia** (absolute lymphocyte counts below 1.2 x 10^9^cells /L) **AND** O_2_Sat ≤ 94% on room air at screening, no oxygen requirement or with an oxygen need of **≤ 2.5 lpm** in nasal cannula.
- **Phase II criteria:** Patients requiring hospitalization with **pneumonia** diagnosed with chest radiograph or computed tomography imaging or **lymphopenia** (absolute lymphocyte counts below 1.2 x 10^9^cells /L) **AND** O_2_Sat ≤ 94% on room air at screening, requiring or not oxygen supplementation (nasal cannula, oxygen mask with reservoir, non-invasive ventilation, etc), but **excluding** mechanical ventilation.
- Have a negative pregnancy test documented prior to enrollment (for females of childbearing potential).
- Be willing and able to comply with study procedures.
- Patients with the ability to comprehend and sign the informed consent
- Written informed consent obtained prior to any screening procedures.

## Exclusion criteria

Patients eligible for this study must not meet any of the following criteria:

- Enrolled in another Clinical Trial for COVID19.
- Rapidly progressive disease with anticipated life-expectancy <72 hours.
- Patients requiring mechanical ventilation.
- Patients with multiorgan failure.
- Moderate - severe (grade ≥ 3) organ impairment (liver, kidney), according to criteria from the National Cancer Institute (NCI CTCAE version 5.0).
- Severe and/or uncontrolled concurrent medical disease that in the opinion of the investigator could cause unacceptable safety risks or compromise compliance with the protocol.
- Have a known history of human immunodeficiency virus infection, Hepatitis B or Hepatitis C; testing is not required in the absence of prior documentation or known history.
- Pregnant or nursing (lactating) women, where pregnancy is defined as the state of a female after conception and until the termination of gestation, confirmed by a positive hCG laboratory test.
- Any other condition that, in the opinion if the Investigator, may interfere with the efficacy and/or safety evaluation of the trial.

1. INVESTIGATIONAL PRODUCT, DOSAGE AND MODE OF ADMINISTRATION

In this clinical trial, our group is proposing the use of adoptive cell therapies for the treatment of COVID-19. Most current clinical trials for the treatment of this disease are investigating the safety and efficacy of antiretroviral or anti-inflammatory drugs.

The two cell therapies proposed in the trial (alloreactive NK cells and memory T cells) are both routinely used in the HSCT setting. These cell subsets are obtained by partial purging of T lymphocytes *ex vivo* using magnetic immunoselection techniques from leukoapheresis material. These procedures are routinely performed as standard of care in the best hemato-oncology centers worlwide. The Cell Therapy and Bone Marrow Unit of the Hematology and Hemotherapy Department at *La Paz* University Hospital, has a wide experience in these cell manipulation techniques, due to the outstanding activity of hematopoietic progenitors transplant carried out in the Hospital.

As examples, figure 4 shows the dynamics of transplant activity over the last 10 years in the Pediatric Hemato-Oncology Department and table 1 shows the different types of HSCT based on the selected cell subpopulations.


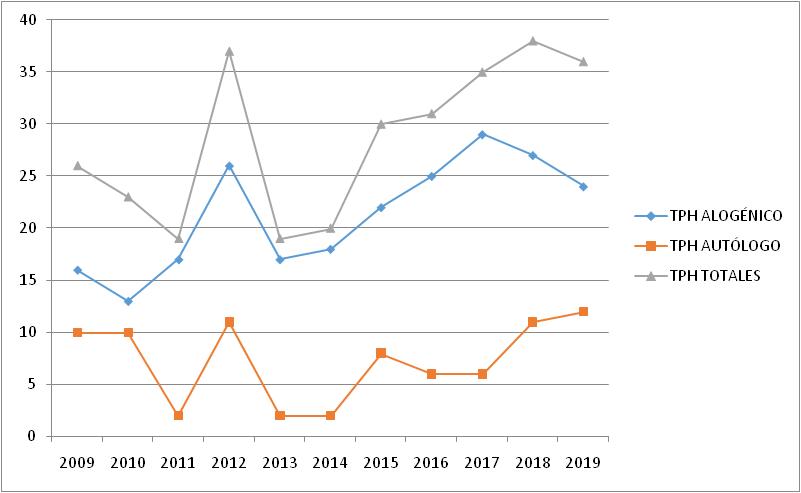


Figure 2: Different types of HSCT performed in the past ten years (Pediatric Hemato-Oncology Department, *La Paz* University Hospital)

Table 1 Number of NK cells and memory T cells infusions in the last two years (Pediatric Hemato-Oncology Department, *La Paz* University Hospital)


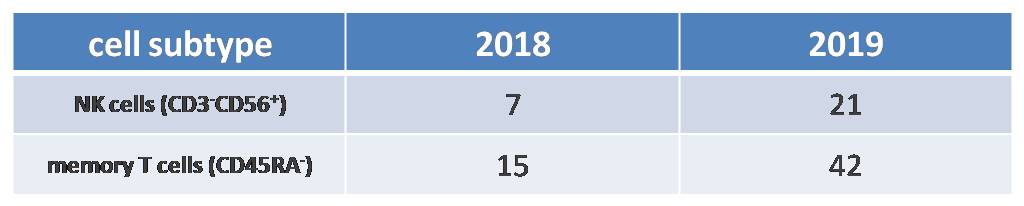


These cell subtype selection procedures are not drugs. They are not investigational medicinal products, and they are not included in the Advanced Therapy Medicinal Products category from the substantial manipulation point of view. This way, the cell therapies included in this trial are regulated as Human Cells and Tissues, within the Directive 2004/23/CE of the European Parliament and Council of 31 March 2004, related to setting standards of quality and safety for the donation, procurement, testing, processing, preservation, storage and distribution of human tissues and cells.

Overall, we believe that the strategies proposed in this trial can contribute to increasing the available therapeutic options in the current emergency due to the novel SARS-CoV-2 coronavirus. Moreover, the production and administration to patients of these products, is also a routine clinical activity for our healthcare team that has been proven essentially safe.

**Investigational Medicinal Product:** memory T cells and NK cells from donors who have recovered from COVID-19 and are currently disease- free.

**Form of administration:** intravenous

**Administration dose and Schedule:**

1. **Single Ascending Dose Cohorts (Phase I)**

All patients will receive the SoC of each site for COVID19. Additionally, they will receive the investigational treatment.

**Arm A:** If one or more HLA class I match between donor and recipient is present, patients will receive memory T cells.

- Cohort 1: starting dose will be up to 1x10^5^/kg.
- Cohort 2: 1x10^5^/kg to 5x10^5^/kg
- Cohort 3: 5x10^5^/kg to 1x10^6^/kg.

**Arm B:** If donor and recipient have less than one HLA class I match or HLA-KIR mismatch, they will receive NK cells.

- Cohort : starting dose will be up to 1x10^6^/kg
- Cohort 2: 1x10^6^/kg to 5x10^6^/kg.
- Cohort 3: 5x10^6^/kg to 1x10^7^/kg.

The next cohort will open if all the patients in the previous cohort have been included and no safety issues have aroused during the follow-up after cell infusion (data of at least 7 days after cell infusion is required to proceed to each subsequent dose).

Those patients showing clinical improvement at day 7 according to the investigator (based on respiratory status and blood-result tests) who haven’t shown previous toxicities of grade 3 or higher, can receive a second cycle with the same dose at Day 7, if the investigator considers it appropriate.

The 18 patients included in the Phase I will continue to be followed for possible DLTs during the 21 days following cell infusion.

1. **Dose level decisions for Phase II** will be guided by safety and DLT data from Phase I. Phase II will begin once RP2D is established based on Phase I data.

The recommended dose will be the maximum tolerated dose (MTD) unless no MTD is determined in the dose escalation segment of the study. In this situation, the recommended dose will be the highest dose evaluated in the dose escalation segment.

Those patients showing clinical improvement at day 7 according to the investigator (based on respiratory status and blood-result tests) who haven’t shown previous toxicities of grade 3 or higher, can receive a second cycle with the same dose at Day 7, if the investigator considers it appropriate.

Third party donors will be individuals in recovery phase, with no symptoms for at least 14 days and at least one SARS-CoV-2 negative test. The blood donor selection will be done by the Regional Blood Transfusion Center. Refer to "donor selection criteria" in section 5 for further details.

Blood donor apheresis will be split into 2 parts, NK cells (CD3-/CD56+) will be selected for Arm A and T memory cells (CD45RA-) for Arm B.

Blood product from one donor can be utilized for more than one patient.

## Subject Treatment Assignment

Patients will be allocated into two groups: Arm A and Arm B depending on HLA typing.

For the phase I, patients on each arm will subsequently undergo a correlative allocation into the different cohorts (1,2,3).

The decision to proceed to each subsequent dose level (next cohort) will be made based on safety and tolerability data from the prior lower dose level. Safety data for at least 7 days post-dose from all the subjects at the prior lower dose will be reviewed before dose escalation.

The next cohort will open if all the patients in the previous cohort have been included and no safety issues have aroused during the follow-up after cell infusion (data of at least 7 days after cell infusion is required to proceed to each subsequent dose). Patients will continue to be followed for possible DLTs during the 21 days following cell infusion.

For phase II, eligible patients will be allocated to arm A or B considering HLA typing and within each arm they will then be randomized in a 1:1 ratio to 1 of 2 treatment groups (SoC or SoC+RP2D).

## Prior and concomitant medication

All patients will receive the SoC of each site for COVID19. Additionally, they will receive the investigational treatment.

SoC medicines and supportive care is permitted according to institutional guidelines. All concomitant medications should be recorded, from the date the informed consent is signed until discharge of the patient.

**Criteria for Discontinuation of Study Treatment**

Subjects may voluntarily withdraw consent to participate in the study for any reason at any time. This will not cause a detriment in their treatment or clinical follow-up.

If a subject discontinues the study, the investigator will try to perform all the needed evaluations to ensure that no adverse event occurs.

## Study medication may be discontinued in the following instances:

- Discharge from the hospital/ institution.
- Concomitant illness that would, in the judgment of the investigator, affect assessments of clinical status to a significant degree.
- Toxicity, or toxicity that, in the judgment of the investigator, compromises the ability to continue study-specific procedures or is considered not to be in the subject’s best interest.
- Subject request to discontinue for any reason.
- Subject's non-compliance.
- Pregnancy during the study.

1. STUDY PROCEDURES AND ASSESSMENTS

The study procedures for each subject enrolled in the study are presented in tabular form in **Table 2** and described in the text that follows.

## Screening Visit

## Subjects will be screened within two days before randomization and dosing to determine eligibility for participation in the study.

- Obtain written informed consent.

After the informed consent is signed, the following assessments will be performed:

- Review SARS-CoV-2 testing.
- Focused medical history including the following information: demographics, date of first symptom, description of symptoms, allergies, concomitant medications and past medical history.
- Targeted physical examination including vital signs (heart rate, temperature, blood pressure), body weight, and height.
- Documentation of respiratory status:
- Respiratory Rate
- SpO2 at rest or PaO2
- Fraction of inspired oxygen (FIO2)
- Oxygen supplementation:
  - nasal cannula
  - high flow oxygen therapy
  - mask with a reservoir bag
  - non-invasive ventilation
- Radiographic findings (chest X ray or chest CT).
- Obtain blood samples if not done in the previous 48 hours for:
- Hematology and coagulation: Hematocrit, Hemoglobin, Mean Corpuscular Hemoglobin Concentration (MCHC), MCV (Mean Corpuscular Volume), Platelets, Red blood cells, White blood cells with differential (Basophils, Eosinophils, Lymphocytes, Monocytes, Neutrophils). **D-Dimer.**

# Chemistry: Glucose (fasting or non-fasting), Urea, Creatinine, Sodium, Potassium, Calcium, Chloride, Total Protein, Albumin, Bilirubin (total and direct), Aspartate Aminotransferase (AST), Alanine Aminotransferase (ALT), Lactate Dehydrogenase (LDH), Ferritin, C-reactive Protein (CRP) , troponin.

# Markers of Immunological function: Immunoglobulins, serum cytokines (IL-6, IL-1, TNFa, IFNb, IL-10); T cell repertoire: CD3 (CD4, CD8), naive CD45RA+CD27+, central memory CD45RA-CD27+, effector memory CD45RA-CD27- y EMRA CD45RA+CD27-, Activated T cells (HLADR+), T regs (CD4+CD25+CD127low), Tgd; NK cells repertoire CD56+CD3- (KIR (2DL1, 2DL2/3, 3DL1), NCR (NKp30, NKp44, NKp46), NKG2D); NKT cells, B cells, memory B cells (CD19+CD27+).

# Additional investigational studies: Blood samples will be stored for research use. These samples will be processed to obtain the cells and a phenotypic study will be carried out to see the recovery in time of immunological markers using the flow cytometry technique. The functionality of infected COVID-19 cells and recovered cells will be studied by co-culture assays to see cytokine secretion by flow cytometry. In addition, plasma cytokines will be studied by protein assays.

- Pregnancy test (for women of childbearing potential).
- Record any serious adverse events and all adverse events related to protocol-mandated procedures occurring after signing the consent form.

Study subjects who fulfil all the inclusion criteria and none of the exclusion criteria should be immediately randomized.

Randomization and dosing should occur on the same day if possible.

## Baseline/Day 0 Assessments

The following evaluations are to be completed at Day 0 visit (infusion date).

The investigator must have confirmed eligibility before proceeding to randomization on the Day 0 visit.

**If the screening and Day 0 visits occur within 24 hours, no procedures need to be repeated.**

Participants must complete the following assessments before administering the study drug (if not done in the previous 24 hours):

- Physical examination including, vital signs (heart rate, temperature, blood pressure, and body weight).
- Documentation of respiratory status:
- Respiratory Rate
- SpO2 at rest or PaO2
- FIO2
- Oxygen supplementation:
  - nasal cannula
  - high flow oxygen therapy
  - mask with a reservoir bag
  - non-invasive ventilation
- Radiographic findings if available.
- Obtain blood samples for:
- Hematology and coagulation: Hematocrit, Hemoglobin, Mean Corpuscular Hemoglobin Concentration (MCHC), MCV (Mean Corpuscular Volume), Platelets, Red blood cells, White blood cells with differential (Basophils, Eosinophils, Lymphocytes, Monocytes, Neutrophils). **D-Dimer.**

# Chemistry: Glucose (fasting or non-fasting), Urea, Creatinine, Sodium, Potassium, Calcium, Chloride, Total Protein, Albumin, Bilirubin (total and direct), Aspartate Aminotransferase (AST), Alanine Aminotransferase (ALT), Lactate Dehydrogenase (LDH), Ferritin, C-reactive Protein (CRP) , troponin.

# Markers of Immunological function: Immunoglobulins, serum cytokines (IL-6, IL-1, TNFa, IFNb, IL-10); T cell repertoire: CD3 (CD4, CD8), naive CD45RA+CD27+, central memory CD45RA-CD27+, effector memory CD45RA-CD27- y EMRA CD45RA+CD27-, Activated T cells (HLADR+), T regs (CD4+CD25+CD127low), Tgd; NK cells repertoire CD56+CD3- (KIR (2DL1, 2DL2/3, 3DL1), NCR (NKp30, NKp44, NKp46), NKG2D); NKT cells, B cells, memory B cells (CD19+CD27+).

# Additional investigational studies: Blood samples will be stored for research use. These samples will be processed to obtain the cells and a phenotypic study will be carried out to see the recovery in time of immunological markers using the flow cytometry technique. The functionality of infected COVID-19 cells and recovered cells will be studied by co-culture assays to see cytokine secretion by flow cytometry. In addition, plasma cytokines will be studied by protein assays.

- Pregnancy test (for women of childbearing potential).
- Record any serious adverse events and all adverse events related to protocol-mandated procedures occurring after signing the consent form.

## Daily Study Assessments

The following evaluations are to be completed daily from Days 1 – 90 or until discharge, whichever occurs earlier (these will be performed as far as possible considering the increased work-load):

- Physical examination including, vital signs (heart rate, temperature, blood pressure).
- Documentation of respiratory status:
- Respiratory Rate
- SpO2 at rest or PaO2
- FIO2
- Oxygen supplementation:
  - nasal cannula
  - high flow oxygen therapy
  - mask with a reservoir bag
  - non-invasive ventilation
  - mechanical ventilation, or ECMO.
- Radiographic findings **if available**.
- **Scales (NEWS and 7-point scale).**
- Review AEs and document concomitant medications.

## Additional Assessments

## Will be performed as far as possible considering the increased work-load and if the investigator considers it appropriate:

- SARS-CoV-2 testing by PCR: Days 7, 14, 21, 28 and 90 or until a negative result is obtained, whichever occurs first.
- Blood samples for:
- Hematology and Chemistry: Day 3, 7, 14, 21, 28.

# Markers of Immunological function: Performed weekly.

- Donor chimerism: Day 3 and 7. If donor chimerism persists by day 7, repeat, 14, 21, 28 or until disappearance (weekly until disappearance).

# Additional investigational studies: Days 3, 7, 14, 21 and 28. Blood samples will be stored for research use. These samples will be processed to obtain the cells and a phenotypic study will be carried out to see the recovery in time of immunological markers using the flow cytometry technique. The functionality of infected COVID-19 cells and recovered cells will be studied by co-culture assays to see cytokine secretion by flow cytometry. In addition, plasma cytokines will be studied by protein assays.

- The first 18 patients will be followed for possible DLTs during the 21 days following cell infusion.

## Schedule of activities phase I

Table 2: Schedule of activities Phase I

| All the visits have a window of +/- 1 day | **Screening/D0** | **D1-D90 or until discharge** | **D7** | **D 14** | **D 21** | **D 28** | **D 90** |
| --- | --- | --- | --- | --- | --- | --- | --- |
| **Informed Consent** | x |  |  |  |  |  |  |
| **SARS-CoV-2 PCR testing** |  |  | x | x | x | x | x |
| **Medical history** | x |  |  |  |  |  |  |
| **Concomitant medications** | x | x | x | x | x | x | x |
| **Physical examination** | x | x | x | x | x | x | x |
| **Vital signs** | x | x | x | x | x | x | x |
| **Respiratory status** | x | x |  | x | x | x | x |
| **Hematology** | x | X^3^(day 3) | x | x | x | x |  |
| **Biochemistry** | x | X^3^(day 3) | x | x | x | x |  |
| **Markers of immunological function^1^** | x |  | x | x | x | x | x |
| **Blood sample for additional investigational studies** | x | X (day 3) | x | x | x | x |  |
| **Donor chimerism** |  | X^2^(day 3) |  | X^2^ | X^2^ | X^2^ |  |
| **Pregnancy test** | x |  |  |  |  |  |  |
| **Scales (NEWS and 7 point scale)** | x | x | x | x | x | x | x |
| **Improvement according to investigator/Second dose**** |  |  | x |  |  |  |  |
| **DLTs** | x | | | | |  |  |
| **Evaluation of patient recovery** |  |  |  | x |  |  |  |
| **Adverse events** | X | | | | | | |

^1^ Performed at screening and weekly. Please review section 7 for specific assessments.

^2^ Days: 3 and 7. If donor chimerism persists by day 7, repeat on days 14, 21, 28 and then continue weekly (until disappearance) Stop once it’s negative.

^3^Day 3

^**^ Those patients showing clinical improvement at day 7 according to the investigator (based on respiratory status and blood-test result) who haven’t shown previous toxicities of grade 3 or higher **and** if donor chimerism doesn´t persist, can receive a second cycle with the same dose at Day 7, if the investigator considers it appropriate.

## Schedule of activities phase II

Table 3: Schedule of activities Phase II

| All the visits have a window of +/- 1 day | **Screening/D0** | **D1-D90 or until discharge** | **D7** | **D 14** | **D 21** | **D 28** | **D 90** |
| --- | --- | --- | --- | --- | --- | --- | --- |
| **Informed Consent** | x |  |  |  |  |  |  |
| **SARS-CoV-2 PCR testing** |  |  | x | x | x | x | x |
| **Medical history** | x |  |  |  |  |  |  |
| **Concomitant medications** | x | x | x | x | x | x | x |
| **Physical examination** | x | x | x | x | x | x | x |
| **Vital signs** | x | x | x | x | x | x | x |
| **Respiratory status** | x | x |  | x | x | x | x |
| **Hematology** | x | X^3^ (day 3) | x | x | x | x |  |
| **Biochemistry** | x | X^3^ (day 3) | x | x | x | x |  |
| **Markers of immunological function^1^** | x |  | x | x | x | x | x |
| **Blood sample for additional investigational studies** | x | X (day 3) | x | x | x | x |  |
| **Donor chimerism** |  | X^2^ (day 3) | x | X^2^ | X^2^ | X^2^ |  |
| **Pregnancy test** | x |  |  |  |  |  |  |
| **Scales (NEWS and 7 point scale)** | x | x | x | x | x | x | x |
| **Evaluation of patient recovery** |  |  |  | x |  |  |  |
| **Improvement according to investigator/Second dose^**^** |  |  | x |  |  |  |  |
| **Adverse events** | x | | | | | | |

^1^ Performed at screening and weekly. Please review section 7 for specific assessments.

^2^ Day: 3 and 7. If donor chimerism persists by day 7, repeat on days 14, 21, 28 and then continue weekly (until disappearance). Stop once it is negative.

^3^Day 3

^**^ Those patients showing clinical improvement at day 7 according to the investigator (based on respiratory status and blood-result tests) who have not shown previous toxicities of grade 3 or higher **and** if donor chimerism doesn´t persist, can receive a second cycle with the same dose at Day 7, if the investigator considers it appropriate.

## Follow-up

Patients will be followed up until day 90, discharge or death, whichever occurs earlier. However, if the patient is discharged before day 30, weekly ambulatory visits (days 7, 14, 21 and 28) will be performed if the investigator considers it appropriate.

Donor chimerism will be evaluated on days specified in the schedule of assessments and weekly until disappearance in all patients.

If the patient has been discharged before day 30 they will be contacted by phone at Day 30 and Day 90 to obtain information about evolution and adverse events.

Patients discharged after day 30 will be contacted by phone at Day 90 to obtain information about evolution and adverse events.

**End of study:** The end of study date will be the date of the last visit of the last patient included.

1. STUDY OUTCOME MEASURES

## Primary outcome:

**Phase I**

The primary outcomes of the Phase I in this study are:

- The primary outcome will be the occurrence of DLTs in all patients during the study treatment, until 21 days after cell infusion, and the MTD.

DLT is defined as:

- - any grade 3 or higher toxicity with an attribution of definitely or probably related to the infusion of the cells and
  - any lower grade toxicity that increases to a grade 3 or higher as a direct result of the cell infusion.

Based on this, **RP2D** will be defined as: The recommended dose will be the MTD unless no MTD is determined in the dose escalation segment of the study. In the latter, the recommended dose will be the highest dose evaluated in the dose escalation segment.

- Incidence and nature of DLT of a single infusion of NK or memory T cells from a healthy donor recovered from COVID-19 (dose escalation).

**Phase II**

The primary outcome in Phase II in this study is:

- The incidence of patient recovery infusing adoptive NK cells or adoptive memory T cells.
- **Recovery is defined as:** Proportion of participants in each group with normalization of fever and oxygen saturation [criteria for normalization: temperature < 38°C armpit, and SpO2 > 94%, sustained for at least 24 hours] or lymphopenia recovery **through Day 14.**

## Secondary outcomes:

- Time (days) to normal level of lymphocytes.
- Proportion of patients showing clinical improvement at day 7 according to the investigator (based on respiratory status and blood-result tests).
- Proportion of patients receiving a second cycle.
- Time (days) to first negative SARS-CoV-2 PCR after infusing adoptive NK cells or adoptive memory T cells.
- The incidence of treatment-related adverse events (new or worsening from baseline) will be summarized by system organ class and/or preferred term, severity, type of adverse event and relation to study treatment. They will be defined by the Common Terminology Criteria for Adverse Events (CTCAE) Version 5.0
- Duration (days) of hospitalization.
- Time (days) to discharge or to a NEWS (Appendix 1) of ≤ 2 and maintained for 24 hours, whichever comes first.
- Time to improvement by one category on a 7-point ordinal scale (Appendix 2).
- Subject clinical status (on a 7-point ordinal scale) at day 14.
- Proportion of patients requiring intensive care unit.
- All-cause mortality at Day 28.

## Exploratory outcomes:

- To determine donor chimerism by short tandem repeats and a variable number of tandem repeat markers using the ABI Prism 3130 System and immune lymphocyte reconstitution by multiparametric flow cytometry after adoptive therapy weekly during the first month after infusion.
- Immune reconstitution: Immunoglobulins, serum cytokines (IL-6, IL-1, TNFa, IFNb, IL-10); T cell repertoire: CD3 (CD4, CD8), naive CD45RA+CD27+, central memory CD45RA-CD27+, effector memory CD45RA-CD27- y EMRA CD45RA+CD27-, Activated T cells (HLADR+), T regs (CD4+CD25+CD127low), Tgd; NK cells repertoire CD56+CD3- (KIR (2DL1, 2DL2/3, 3DL1), NCR (NKp30, NKp44, NKp46), NKG2D); NKT cells, B cells, memory B cells (CD19+CD27+).

#

1. SAFETY MONITORING AND REPORTING

## DEFINITIONS

**Adverse event (AE):**

Any untoward occurrence harmful to the health of a patient or clinical trial subject treated with a medicinal product and which does not necessarily have a causal relationship with the study treatment.

Abnormal laboratory values or test results constitute AEs only if they fulfill at least one of the following criteria:

- they induce clinical signs or symptoms
- they are considered clinically significant
- they require treatment

**Adverse Reaction (AR):**

An AR is any harmful and unintended reaction to an investigational medicinal product, regardless of the dose administered.

**Serious Adverse Event (SAE) and Serious Adverse Reaction (SAR):**

A serious adverse event is any untoward medical occurrence that at any dose:

- Results in death,
- Is life-threatening (NOTE: The term "life-threatening" in the definition of "serious" refers to an event in which the participant was at risk of death at the time of the event; it does not refer to an event which hypothetically might have caused death if it were more severe),
- Requires inpatient hospitalization or prolongation of existing hospitalization,
- Results in persistent or significant disability/incapacity, or
- Is a congenital anomaly/birth defect.
- Other important medical events. NOTE: Other events that may not result in death are not life threatening, or do not require hospitalization, may be considered a serious adverse event when, based upon appropriate medical judgment, the event may jeopardize the patient and may require medical or surgical intervention to prevent one of the outcomes listed above.

The concept "requires hospitalisation" would exclude both hospitalisation planned for scheduled treatment as well as those that were planned or envisaged before the study started in connection with a pre-existing medical condition.

All malignant neoplasms will be assessed as serious under “medically significant” if other serious criteria are not met and the malignant neoplasm is not a disease progression of the study indication.

Any suspected transmission via a medicinal product of an infectious agent is also considered a serious adverse reaction.

## CLASSIFICATION OF AN ADVERSE EVENT

**Severity of an Event**

Adverse events will be assessed and graded according to the Common Terminology Criteria for Adverse Events (CTCAE) version 5.0. If CTCAE grading does not exist for an AE, the severity of mild, moderate, severe, life-threatening and fatal, corresponding to Grades 1-5, will be used.

**Relationship to Study INTERVENTION**

All adverse events (AEs) must have their relationship to study intervention assessed by the clinician who examines and evaluates the participant based on temporal relationship and his/her clinical judgment. The degree of certainty about causality will be graded using the categories below. In a clinical trial, the study product must always be suspected.

- **Related** – The AE is known to occur with the study intervention, there is a reasonable possibility that the study intervention caused the AE, or there is a temporal relationship between the study intervention and event. Reasonable possibility means that there is evidence to suggest a causal relationship between the study intervention and the AE.
- **Not Related** – There is not a reasonable possibility that the administration of the study intervention caused the event, there is no temporal relationship between the study intervention and event onset, or an alternate etiology has been established.

**Expectedness**

Investigators will be responsible for determining whether an adverse event (AE) is expected or unexpected. An AE will be considered unexpected if the nature, severity, or frequency of the event is not consistent with the risk information previously described for the study intervention.

**Unexpected adverse reaction:**

An adverse reaction, the nature or severity of which is not consistent with the applicable product information.

**Suspected Unexpected Serious Adverse Reaction (SUSAR):**

Serious adverse drug reaction (SAR) that is unexpected or for which the development is uncommon (unexpected issue) observed during a clinical trial and for which there is a relationship with the study drug.

These adverse drug reactions, characterized to be serious and unexpected, need to be communicated immediately (mandatory) in accordance with national legislation and international guidelines.

## INFORMATION ON ADVERSE EVENTS

The investigator will monitor and systematically collect the AEs starting from the signing of the Informed Consent Form until the final follow-up visit of each subject.

The occurrence of AEs must be sought by non-directive questioning of the subject at each visit during the study. Adverse events may also be detected through physical examination findings, laboratory test findings, or other assessments.

Also, the investigator will assess and record, in detail, the AE, including the start and end dates, the description of the event, severity, course, outcome, the relationship of the AE with the investigational medicinal product and the measures taken (treatments, additional complementary examinations, etc.).

The AE will be recorded in the medical history and recorded in the Case Report Form.

The subjects who present with an AE will be subjected to relevant follow-up until its resolution or until it is judged to be permanent. The type of follow-up will be conducted according to the relevant criteria depending on the particular event.

## SAE REPORTING

If an SAE is produced, the investigator shall notify the sponsor or whoever assumes the tasks delegated by the sponsor, within a maximum period of 24 hours from the time of knowing about the event.

Also, the investigator shall complete and sign the SAE report form and send it by fax or email to:

**irene.ucicec @gmail.com**

**Person Responsible For Pharmacovigilance**

**Unidad Central de Investigación Clínica y Ensayos Clínicos (UCICEC)**

**Hospital Universitario La Paz**

**Paseo de la Castellana, 261**

**28046 Madrid**

**Tel: 91 207 14 66/Fax: 91 207 14 66**

The sponsor or whoever assumes the tasks delegated by the sponsor will review the received form and, if necessary, request additional information from the investigator. The investigator will provide information to the sponsor or whoever assumes the tasks delegated by the sponsor, whenever asked and, in all cases, when the initial assessment changes in severity or causality. To communicate the follow-up information, repeat the reporting procedure described previously.

The sponsor, or whoever assumes the tasks delegated by the sponsor, shall keep detailed records of all the SAEs or the events of special interest, which are notified by the investigators.

In the case of a medication error or if the investigational medicinal product is used outside the provisions of the protocol, while conducting the study, the investigator shall notify the sponsor or whoever assumes the tasks delegated by the sponsor, within 24 hours from the time of knowing about the event. The reporting circuit and form will be the same as for the SAE.

**SUSAR EXPEDITED REPORTING**

The sponsor, or whoever assumes the tasks delegated by the sponsor, will notify all suspected SUSARs, to the Ethics Committee within a period of fifteen calendar days from the time of knowing about the event. When the SUSAR has caused the death of the patient or has been life-threatening, the notification shall be made within a maximum period of seven calendar days from the time of knowing about the event. The relevant information concerning subsequent events will be filled in within the following eight days.

## EXPEDITED REPORTING OF OTHER RELEVANT SAFETY INFORMATION

The sponsor, or whoever assumes the tasks delegated by the sponsor, shall notify, as soon as possible and no later than 15 days after having knowledge of it, any information that could alter the benefit/risk relationship of the investigational medicinal product (e.g. an increase in the rate of occurrence of the expected SAR, SUSARs that occur after the completion of a clinical trial, new events related to the conduct of the trial or the development of the investigational medicinal product, any recommendation of the Data Monitoring Committee where relevant for the safety of subjects, etc.

## PREGNANCY REPORTING

Subjects will be instructed to notify the investigator if the subject or partner becomes pregnant.

If any pregnancy occurs during the course of the study, the investigator shall notify the sponsor, or whoever assumes the tasks delegated by the sponsor, within 24 hours of knowing about the event. Also, a follow-up of the pregnancy will be performed to document its outcome and the state of health of the newborn. If the pregnancy outcome meets the SAE criteria or if the newborn presents a serious event, the procedures for reporting an SAE will be followed.

1. **ETHICAL CONSIDERATIONS**

## General Considerations

This clinical study was designed and shall be implemented, executed and reported in accordance with the ICH Harmonized Tripartite Guidelines for Good Clinical Practice, with applicable local regulations ([Law 14/2007](http://www.boe.es/buscar/doc.php?id=BOE-A-2007-12945), July 3^rd^, on Biomedical Research (*Ley de Investigación Biomédica*, BOE 159 of July 4^th^, 2007 and the DIRECTIVE 2004/23/EC OF THE EUROPEAN PARLIAMENT AND OF THE COUNCIL of 31^st^ March 2004 on setting standards of quality and safety for the donation, procurement, testing, processing, preservation, storage and distribution of human tissues and cells) and with the ethical principles laid down in the Declaration of Helsinki.

Before initiating the trial, the investigator/ institution must obtain approval/favorable opinion from the Independent Ethics Committee for the trial protocol, written informed consent form, consent form updates, subject recruitment procedures and any other written information to be provided to subjects.

Prior to the study start, the investigator is required to sign a protocol signature page confirming his/ her agreement to conduct the study in accordance with these documents and all of the instructions and procedures found in this protocol.

Neither patients nor researchers will receive remuneration for their participation in the study.

## Informed Consent

Informed consent must be obtained before conducting any study-specific procedures.

Eligible subjects will be informed before the beginning of the study about the objectives and procedures, as well as the potential risks derived from his/ her study participation.

The process of obtaining informed consent must be documented in the subject source documents.

Women of child bearing potential must be informed that taking the study treatment may involve unknown risks to the fetus if pregnancy were to occur during the study and agree that in order to participate in the study they must adhere to the contraception requirements. Male subjects must be informed that if a female partner becomes pregnant while he is enrolled in the study, contact with the female partner will be attempted to request her consent to collect pregnancy outcome information.

## Data management, confidentiality of trial documents and patients’ records.

The information obtained in this study will be confidential and treated at all times as such.

The handling, communication and transfer of personal data of all the subjects participating in the study will be protected, complying with the basic ethical principles of Biomedical Research and with the applicable regulations: Regulation (EU) 2016/679 of the European Parliament and of the Council of 27^th^ April 2016 on the protection of natural people with regard to the processing of personal data and on the free movement of such data, and repealing Directive 95/46/EC (General Data Protection Regulation) and LAW 41/2002, of 14^th^ November, regulating patient autonomy and rights and obligations of information and clinical documentation.

A CRF must be completed for all patients that have given informed consent.

All entries into the CRF are the responsibility of the investigator or a qualified designated staff member.

Each subject is identified in the study by a Subject Number (Subject No.) that is assigned when the subject is first enrolled for screening and is retained as the primary identifier for the subject throughout his/ her entire participation in the trial. Once assigned, this number must not be reused for any other subject.

Trial records and source documents, including signed ICF, pertaining to the conduct of this study must be retained by the investigator for 25 years from the issue of the final Clinical Study Report/ equivalent summary, in accordance with the applicable regulations (Royal Decree 1090/2015, of 4^th^ December, regulating clinical trials with medicinal products, Ethics Committees for Investigation with medicinal products and the Spanish Clinical Studies Registry).

## Insurance

Insurance coverage is provided for the clinical study.

1. **PRACTICAL CONSIDERATIONS**

## Responsibilities Of Patients

Participants should follow the indications of the Investigator and communicate any eventuality to them. Subjects will be informed of the restrictions that they must adhere to during the trial.

Subjects may voluntarily withdraw consent to participate in the study for any reason at any time. This will not cause a detriment in their clinical follow-up.

If a subject discontinues the study, the investigator will try to ensure that all the needed evaluations to ensure that no adverse event occurs are performed.

## Responsibilities Of The Investigator

Investigators will work in accordance with the national legislation.

Prior to the study start, the investigator is required to sign a protocol signature page confirming his/ her agreement to conduct the study in accordance with these documents and all of the instructions and procedures found in this protocol and to give access to all relevant data and records to monitors, auditors, ECs, and regulatory authorities as required.

## Protocol Adherence

Investigators ascertain they will apply due diligence to avoid protocol deviations. If deviations occur, the investigator must inform the monitor and the consequences of such deviations will be reviewed and discussed among the team. All protocol deviations will be documented/ recorded specifying reason, data, action taken, and consequences in patients and in the study. All documentation related to deviation will be stored in the Investigator File.

## Monitorization, Audit and Inspection

The study will be monitored by SCReN. An appropriate monitoring plan for this study has been developed.

The monitor will visit the site to check the completeness of subject records, the accuracy

of data capture/ data entry, the adherence to the protocol and to Good Clinical Practice, the progress of enrollment, and to ensure that study treatment is being stored, dispensed, and accounted for according to specifications.

The investigator must give the monitor access to all relevant source documents for monitoring activities, audits, EC revisions, and inspections from Health Authorities.

The investigator must maintain source documents for each subject in the study, consisting of case and visit notes (hospital or clinical medical records) containing demographic and medical information, laboratory data, electrocardiograms, and the results of any other tests or assessments. All information on CRFs must be traceable to these source documents in the subject’s file. The investigator must also keep the original informed consent form signed by the subject (a signed copy should be given to the subject).

# Publication Of The Study Protocol And Results

The protocol will be registered in a publicly accessible database. In addition, after study completion and finalization of the study report, the results of this trial will be submitted for publication in a scientific journal or will be made public, complying with the Declaration of Helsinki:

“Researchers, authors, sponsors, editors and publishers all have ethical obligations with regard to the publication and dissemination of the results of research. Researchers have a duty to make publicly available the results of their research on human subjects and are accountable for the completeness and accuracy of their reports. All parties should adhere to accepted guidelines for ethical reporting. Negative and inconclusive as well as positive results must be published or otherwise made publicly available. Sources of funding, institutional affiliations and conflicts of interest must be declared in the publication. Reports of research not in accordance with the principles of this Declaration should not be accepted for publication”.

# Protocol Amendments

The Principal investigator will ensure that the study protocol is strictly adhered to throughout, and that all data are collected and recorded correctly on the CRF.

All protocol modifications must be documented in writing. Any protocol amendment will be identified by a consecutive number, and must be approved and signed by the sponsor and the Principal investigator.

If amendments are relevant, approval by EC must occur before any changes can be implemented.

When the change involves only logistical or administrative aspects of the trial [e.g. change in clinical monitor[s], change of telephone number[s], the EC must be notified.

Amendments that are required for subject safety may be implemented immediately provided the health authorities are subsequently notified by protocol amendment and the reviewing EC is notified.

The protocol amendment can be initiated by either sponsor or by any Principal investigator.

# Ethics Committee (EC)

Before initiating a clinical trial, the investigator/institution must obtain approval from the Ethics Committee in regards to trial protocol, written informed consent form, consent form updates, subject recruitment procedures (eg: advertisements) and any other written information to be provided to subjects.

The ethics committee decision will be provided in writing to the investigator, a copy will be sent to the Sponsor.

The Sponsor will send periodic reports about the clinical trial conduct to the EC, as well as all suspected unexpected serious adverse reactions. The Sponsor will notify the end of the study to the Ethics Committee.

1. **DATA ANALYSIS AND STATISTICAL METHODS**

# General considerations

Tabular summaries will be presented by treatment group.

Categorical data will be summarized by number and percentages. Continuous variables will be summarized by descriptive statistics. Recover incidence (RI): Time to recovery.

The overall survival rate will be calculated by the nonparametric Kaplan-Meier method. Fisher exact test will be used to identify those patient characteristics that are significantly associated with treatment response (complete or partial).

For quantitative characteristics a chi-squared trend test will be conducted to examine whether the proportion of patients responding to treatment either increased or decreased across the levels of the characteristic.

The results of the statistical analysis will be displayed in the tables and as mean ± SD in the Figures. Levels of significance will be expressed as p-values (*p < 0.05).

# Sample size

A total of **182** patients will be included in this clinical trial.

The plan is to enroll **18** patients in the first dose escalation segment (phase I): **9** patients **per arm** (**3** patients **in each cohort** for both arms).

A total of **164** patients will be included in the phase II: **82** patients **per arm**.**BIBLIOGRAPHY**

- 1. Jones DS. History in a Crisis - Lessons for Covid-19 [published online ahead of print, 2020 Mar 12]. N Engl J Med. 2020;10.1056/NEJMp2004361. doi:10.1056/NEJMp2004361
  2. Guan WJ, Ni ZY, Hu Y, et al. Clinical Characteristics of Coronavirus Disease 2019 in China [published online ahead of print, 2020 Feb 28]. N Engl J Med. 2020;10.1056/NEJMoa2002032. doi:10.1056/NEJMoa2002032
  3. Dong L, Hu S, Gao J. Discovering drugs to treat coronavirus disease 2019 (COVID-19). DrugDiscovTher. 2020;14(1):58–60. doi:10.5582/ddt.2020.01012
  4. Wang M, Cao R, Zhang L, et al. Remdesivir and chloroquine effectively inhibit the recently emerged novel coronavirus (2019-nCoV) in vitro. Cell Res. 2020;30(3):269–271. doi:10.1038/s41422-020-0282-0
  5. Xia W, Shao J, Guo Y, Peng X, Li Z, Hu D. Clinical and CT features in pediatric patients with COVID-19 infection: Different points from adults [published online ahead of print, 2020 Mar 5]. PediatrPulmonol. 2020;10.1002/ppul.24718. doi:10.1002/ppul.24718
  6. Dong Y, Mo X, Hu Y, et al. Epidemiological Characteristics of 2143 Pediatric Patients With 2019 Coronavirus Disease in China [published online ahead of print, 2020 Mar 16]. Pediatrics. 2020;e20200702. doi:10.1542/peds.2020-0702
  7. Willan J, King AJ, Hayes S, Collins GP, Peniket A. Care of haematology patients in a COVID-19 epidemic [published online ahead of print, 2020 Mar 15]. Br J Haematol. 2020;10.1111/bjh.16620. doi:10.1111/bjh.16620
  8. Rizzo R, Di Luca D. Human herpesvirus 6A and 6B and NK cells.ActaMicrobiolImmunol Hung. 2018;65(2):119–125. doi:10.1556/030.65.2018.010
  9. Flórez-Álvarez L, Hernandez JC, Zapata W. NK Cells in HIV-1 Infection: From Basic Science to Vaccine Strategies.Front Immunol. 2018;9:2290. Published 2018 Oct 17. doi:10.3389/fimmu.2018.02290
  10. Mikulak J, Oriolo F, Zaghi E, Di Vito C, Mavilio D. Natural killer cells in HIV-1 infection and therapy. AIDS. 2017;31(17):2317–2330. doi:10.1097/QAD.0000000000001645
  11. Herzig E, Kim KC, Packard TA, et al. Attacking Latent HIV with convertibleCAR-T Cells, a Highly Adaptable Killing Platform. Cell. 2019;179(4):880–894.e10. doi:10.1016/j.cell.2019.10.002
  12. NK cells treatment for novel coronavirus pneumonia. ClinicalTrials.gov Identifier: NCT04280224
  13. Kondo T, Imura Y, Chikuma S, et al. Generation and application of human induced-stem cell memory T cells for adoptive immunotherapy. CancerSci. 2018;109(7):2130–2140. doi:10.1111/cas.13648
  14. Busch DH, Fräßle SP, Sommermeyer D, Buchholz VR, Riddell SR. Role of memory T cell subsets for adoptive immunotherapy. SeminImmunol. 2016;28(1):28–34. doi:10.1016/j.smim.2016.02.001
  15. Maschan M, Blagov S, Shelikhova L, et al. Low-dose donor memory T-cell infusion after TCR alpha/beta depleted unrelated and haploidentical transplantation: results of a pilot trial. Bone Marrow Transplant. 2018;53(3):264–273. doi:10.1038/s41409-017-0035-y
  16. Perruccio K, Sisinni L, Perez-Martinez A, et al. High Incidence of Early Human Herpesvirus-6 Infection in Children Undergoing Haploidentical Manipulated Stem Cell Transplantation for Hematologic Malignancies. BiolBloodMarrowTransplant. 2018;24(12):2549–2557. doi:10.1016/j.bbmt.2018.07.033
  17. Fernández L, Fernández A, Mirones I, et al. GMP-Compliant Manufacturing of NKG2D CAR Memory T Cells Using CliniMACS Prodigy. Front Immunol. 2019;10:2361. Published 2019 Oct 10. doi:10.3389/fimmu.2019.02361
  18. Fernández L, Leivas A, Valentín J, et al. How do we manufacture clinical-grade interleukin-15-stimulated natural killer cell products for cancer treatment?.Transfusion. 2018;58(6):1340–1347. doi:10.1111/trf.14573
  19. Vela M, Corral D, Carrasco P, et al. Haploidentical IL-15/41BBL activated and expanded natural killer cell infusion therapy after salvage chemotherapy in children with relapsed and refractory leukemia. CancerLett. 2018;422:107–117. doi:10.1016/j.canlet.2018.02.033
  20. Vela M, Del Rosal T, Pérez-Martínez A, et al. Possible role of highly activated mucosal NK cells against viral respiratory infections in children undergoing haematopoietic stem cell transplantation.Sci Rep. 2019;9(1):18792. Published 2019 Dec 11. doi:10.1038/s41598-019-55398-y
  21. Pérez-Martínez A, Fernández L, Valentín J, et al. A phase I/II trial of interleukin-15--stimulated natural killer cell infusion after haplo-identical stem cell transplantation for pediatric refractory solid tumors. Cytotherapy. 2015;17(11):1594–1603. doi:10.1016/j.jcyt.2015.07.011
  22. Ng Y, Li Z, Chua YX, et al. Evaluation of the Effectiveness of Surveillance and Containment Measures for the First 100 Patients with COVID-19 in Singapore - January 2-February 29, 2020. MMWR Morb Mortal Wkly Rep. 2020;69(11):307–311. Published 2020 Mar 20. doi:10.15585/mmwr.mm6911e1
  23. Siddiqi, Hasan K. et al. COVID-19 Illness in Native and Immunosuppressed States: A Clinical-Therapeutic Staging Proposal.TheJournal of Heart and LungTransplantation, Volume 0, Issue 0
  24. Mehta P, McAuley DF, Brown M, et al. COVID-19: consider cytokine storm syndromes and immunosuppression [published online ahead of print, 2020 Mar 16]. Lancet. 2020;S0140-6736(20)30628-0. doi:10.1016/S0140-6736(20)30628-0
  25. National Early Warning Score (NEWS) 2.Disponible en: <https://www.rcplondon.ac.uk/projects/outputs/national-early-warning-score-news-2>.
  26. Requirement of FDA for the use of plasma therapy in COVID-19: Available at: https://www.fda.gov/vaccines-blood-biologics/investigational-new-drug-ind-or-device-exemption-ide-process-cber/investigational-covid-19-convalescent-plasma-emergency-inds

# APPENDIX 1. The NEWS scoring system^24^

The NEWS scoring system is based on a simple aggregate scoring system in which a score is allocated to physiological measurements, already recorded in routine practice, when patients present to, or are being monitored in hospital. Six simple physiological parameters form the basis of the scoring system:

1. respiratory rate
2. oxygen saturation
3. systolic blood pressure
4. pulse rate
5. level of consciousness or new confusion*
6. temperature.

**The patient has new-onset confusion, disorientation and/or agitation, where their mental state was previously normal – this may be subtle. The patient may respond to questions coherently, but there is some confusion, disorientation and/or agitation. This would score 3 or 4 on the Glasgow Coma Score (rather than the normal 5 for verbal response), and scores 3 on the NEWS system.*

A score is allocated to each parameter, with the magnitude of the score reflecting how extremely the parameter varies from the normal value. The score is then aggregated and uplifted by 2 points for people requiring supplemental oxygen to maintain their recommended oxygen saturation.


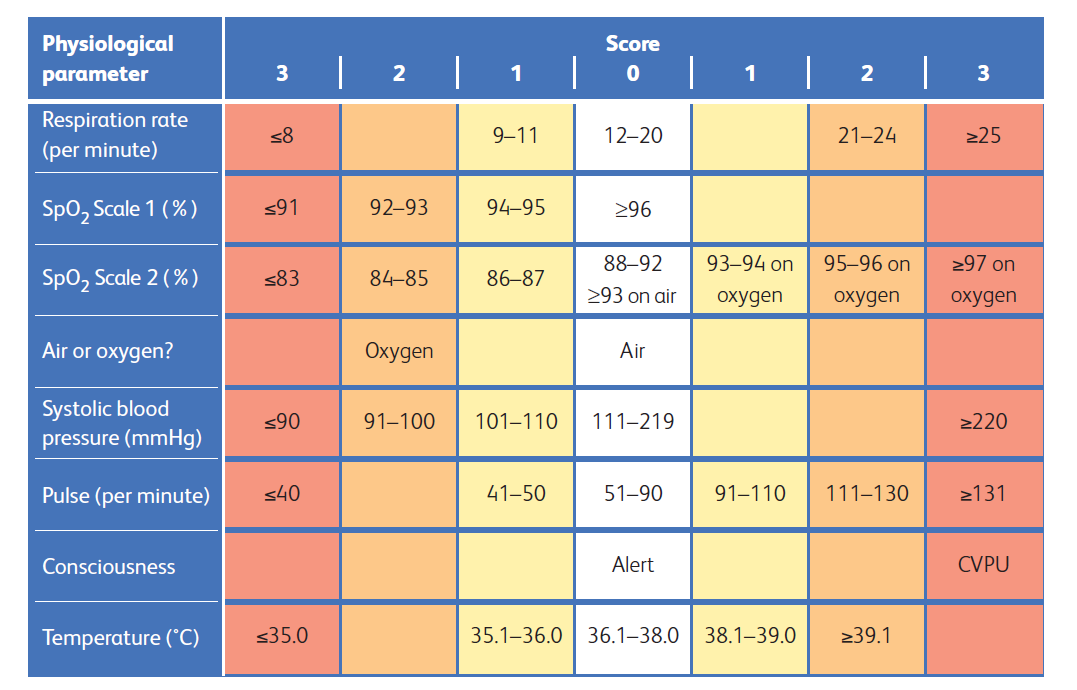


# Appendix 2: 7-point Ordinal Scale for Clinical Improvement

1. Discharged (or “ready for discharge” as evidenced by normal body temperature and

respiratory rate, and stable oxygen saturation on ambient air or > 2L supplemental oxygen)

2. Non-ICU hospital ward (or “ready for hospital ward”) not requiring supplemental oxygen

3. Non-ICU hospital ward (or “ready for hospital ward”) requiring supplemental oxygen

4. ICU or non-ICU hospital ward, requiring non-invasive ventilation or high-flow oxygen

5. ICU, requiring intubation and mechanical ventilation

6. ICU, requiring ECMO or mechanical ventilation and additional organ support (e.g.

vasopressors, renal replacement therapy)

7. Death

# APPENDIX 3: Declaration of Helsinki

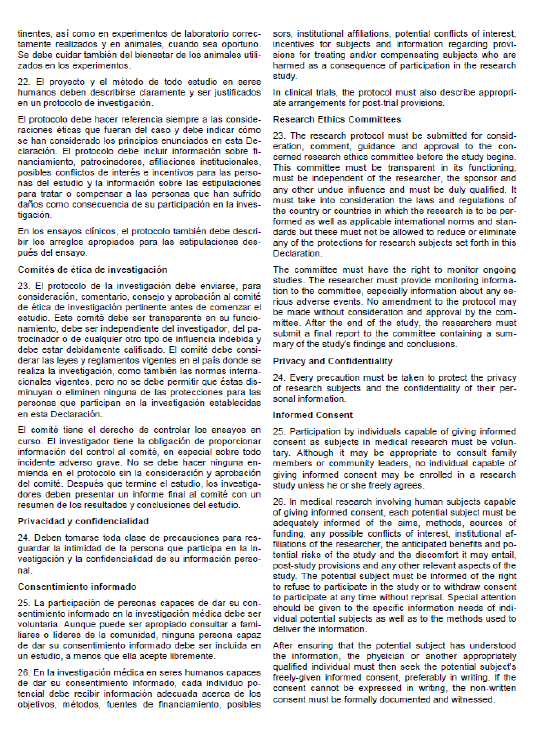

#
