# Supplementary material for: A phase I/II dose-escalation multi-center study to evaluate the safety of infusion of natural killer cells or memory T cells as adoptive therapy in coronavirus pneumonia and/or lymphopenia: RELEASE study protocol
Source: Trials. 2021 Oct 2;22:674. doi: 10.1186/s13063-021-05625-7 (PMC8487326; doi:10.1186/s13063-021-05625-7)
Supplement: Supplementary file 2 — Additional file 2. Informed Consent v4.0 18th March 2021. [file 13063_2021_5625_MOESM2_ESM.docx]

**INFORMATION SHEET FOR PATIENT AND DOCUMENT OF**

**INFORMED CONSENT**

**Study title:** Phase I / II, multicenter, dose escalation clinical trial to evaluate the safety of an infusion of Natural Killer cells or memory T cells as adoptive therapy in coronavirus pneumonia and / or lymphopenia.

**Phase II of the study. Safety and effectiveness phase**

**Study code**: RELEASE

**Study promoter**: Dr. Antonio Pérez Martínez

**Coordinating researcher**: Dr. Antonio Pérez Martínez

**Center address**:

**INTRODUCTION**

We are writing to you to inform you about a research study in which you are invited to participate. The study has been approved by a Research Ethics Committee following current legislation, Law 14/2007, of July 3, on Biomedical Research. The cell therapy to be used in this study belongs to the human cells and tissues category, so it is regulated within the Directive 2004/23 / EC of the European Parliament and of the Council of March 31, 2004, framed in the quality and safety models for the donation, acquisition, testing, processing, preservation, storage and distribution of human cells and tissues.

We intend that you receive correct and sufficient information so that you can decide whether participating or not in this study. For that purpose, you should read this information sheet carefully and ask any questions you have. In addition, you can consult with the people you deem appropriate.

**VOLUNTARY PARTICIPATION**

We invite you to participate in this study because you have an infection caused by the SARS-CoV-2 virus, a disease that was first identified in China in December 2019 and is known as COVID-19. You should know that your participation in this study is voluntary and that you can decide NOT to participate. If you decide to participate, you can change your decision and withdraw your consent at any time, without thereby altering your relationship with your doctor or causing any harm to your health care.

**WHAT IS THE OBJECTIVE OF THIS STUDY?**

The first cases of COVID-19 were detected in Wuhan (China), in December 2019. The disease is caused by a new virus that is transmitted between people who maintain close contact with each other or through droplets produced by coughing or sneezing. About 80% of patients with the disease improve without the need for hospital treatment, but the rest contract a serious illness with a poor prognosis. Currently, there are no medications that have been shown to improve the disease.

The cases with the worst prognosis have an alteration in the functioning of the immune system. These patients have decreased blood cells named lymphocytes and NK cells, which are the cells that act as a defense against the virus, so carrying out an intervention on the immune system seems that could improve the course of the disease. This consists of injecting cells from a donor who has recovered from the SARS-CoV-2 infection, the same that you suffer from. The procedure is known as cell therapy, which has already been used with success in other diseases.

This injection of blood cells from a donor who has already been in contact with the virus would create a faster and stronger response to infection in patients who have the disease. The purpose of this study is to determine whether the infusion of donor NK cells and T lymphocytes to a patient with SARS-CoV-2 virus infection could be effective for the treatment of the disease, compared with patients receiving only the treatment that is used in the hospital for all patients with their disease (standard treatment), since there is no clinical evidence for it yet.

**WHAT DOES THIS STUDY CONSIST OF?**

Your doctor will record your personal data and decide if you have any contraindication to participate in the study, which includes the participation of 164 subjects, in a total of 5 different health centers.

This is a study in which patients with the disease caused by the SARS-CoV-2 virus, known as COVID19, the same disease that you suffer, will participate. It will be used a therapy named cell therapy, where cells that act against the virus will be removed from the blood of people who have had the SARS-CoV-2 virus infection and have recovered. These people have cells of their immune system in their blood that have already been in contact with the virus, being able to create stronger immune responses when injected into patients with the disease.

All participants in this study will receive the standard treatment. Half of them will receive only this standard treatment, and the other half will also receive the infusion of cells. You will be randomly assigned to receive the standard treatment or the standard treatment plus cells, meaning that the treatment groups are assigned randomly. Neither you nor your treating doctor will be able to decide whether or not to receive the cells, and you have a 50% chance of receiving them or not.

In patients assigned to receive the cells, NK cells or T lymphocytes will be received based on how well your blood matches with your donor blood.

- NK cells are cells that act quickly, being the first line of defense against viruses.
- Memory T lymphocytes appear a while after a viral infection occurs. They have long-term memory, so that, when faced with a new infection by the same virus, they offer stronger protection.

The amount of cells to receive has been defined in a study carried out previously, in patients with the same disease, in which 3 different doses of both T and NK cells have been tested. The dose that has been considered the most suitable has been selected among these three different doses.

Participants who have been assigned to receive the cells will be then assigned to two different treatment groups according to the results of the study of the biology of their blood and the blood of their donor. They will receive NK cells or T lymphocytes depending on the compatibility between the cells of their blood and the blood of the donor.

In summary, if you meet all the requirements and criteria to participate in the study, and according to the compatibility characteristics between you and your donor, you will be assigned to one of the following groups:

- Group 1: The standard treatment in which patients with the same disease are treated.
- Group 2: The standard treatment in which patients with the same disease are treated + cell therapy.

If you belong to group 2, you can receive NK cells or T lymphocytes depending, as we have already said, on the results of the study of the biology of your blood and the blood of your donor.

**STUDY ACTIVITIES**

The duration of your participation in the study will be 3 months. If you belong to the group that includes treatment with cells, the dose of NK cells or memory T cells will be infused intravenously, depending on the characteristics of compatibility with your donor. If you are not in the group that includes cell treatment, you will only be administered the standard treatment that is being given to all patients with COVID19.

An initial screening visit will be conducted before you are assigned to a treatment group, where a complete physical examination will be performed and your medical history, personal history, and previous illnesses will be reviewed to verify that you are eligible to participate in the study. Blood samples will also be drawn for a complete blood test, as well as specific determinations that indicate how well your immune system is working. Blood will also be drawn to extract cells and freeze them to investigate how cells infected by coronavirus work and their interaction with the immune system in the future. In women of childbearing age, a pregnancy test will be done. Blood tests will be repeated on day 3 and weekly. If less than 24 hours pass between the screening visit and visit 0, it will not be necessary to perform all the above procedures again. If more than 24 hours have elapsed, the physical examination, complete blood tests and markers of immune function should be repeated.

After the visit, an attempt will be made to the extent possible, depending on the care load, to perform a daily physical examination and an evaluation of your respiratory status, as well as a chest X-ray when your doctor deems it necessary. Blood samples will be taken for analysis, as far as possible and always taking into account the care burden, on days 3, 7, 14 and weekly until day 28. If you belong to the group that receives the treatment with cells, the dose of the treatment under investigation will be infused on the day of visit 0. However, a second additional infusion with the same dose as the first could be given at your discretion 7 days after receiving the first dose from your doctor, if the treatment is working.

Follow-up will be done until day 90 from the infusion of the treatment or until discharge from hospital, whichever occurs sooner. However, if you are discharged before the 28th, you will have to come to the hospital for visits on the 7th, 14th, 21st and 28th from the infusion if your doctor considers it necessary and you will be contacted by phone on the 30th and 90th. If you are discharged after the 30th day, you will be contacted only on the 90th day.

The table below indicates what will happen each day, if possible, taking into account the current care load.

|  | Informed consent | COVID-19 test | Physical exploration | Blood test | Picture test |
| --- | --- | --- | --- | --- | --- |
| Selection visit | X | X | X | X | X |
| Day 0 |  |  | X | X | X |
| Day 1-Day 90 or until discharge |  |  | X |  | X |
| Day 3 |  |  | X | X | X |
| Day 7 |  | X | X | X | X |
| Day 14 |  | X | X | X | X |
| Day 21 |  | X | X | X | X |
| Day 28 |  | X | X | X | X |
| Day 90 |  | X | X | X |  |

Feel free to ask your study doctor if you have questions regarding the procedures and tests that are performed at each visit.

**RISKS AND HAZARDS DERIVED FROM YOUR PARTICIPATION IN THE STUDY**

The investigational product, that is, NK cells and T lymphocytes, used in this study for your disease, is under research. However, the infusion of these kinds of cells has already been used in other diseases, observing an adequate safety margin.

Possible risks that you may experience from the procedures in this study are listed below:

1. Risks of NK cell and T lymphocyte infusion: Side effects are rare and may include:

- Infusion reaction (Cytokine release syndrome: symptoms similar to a flu syndrome (fever, chills, nausea or vomiting, diarrhea, muscle and joint pain).
- Worsening of your respiratory distress.
- If the product to be administered is frozen, you may experience redness, itching, headache, fever, nausea or vomiting, abdominal cramps, diarrhea, high or low blood pressure, changes in heart rate, discomfort in the chest, cough, or shortness of breath. To prevent these effects, in case the product is frozen, medication will be administered before the infusion of the cells.
- Graft versus host disease (GVHD): occurs when the donor's immune system attacks the recipient, and can affect the skin, liver, and digestive system in the acute phase, and can become chronic, causing dry mouth or eye and skin dryness. The percentage of this complication is expected to be very low, less than 1%.

1. Risks derived from taking samples and analytics: all samples and analytics to be performed consist of blood tests that may cause slight discomfort or bruising at the extraction site.

**POSSIBLE BENEFITS**

Your participation in this study may or may not provide you with some benefit in terms of recovery from your illness. However, all the knowledge derived from this study will allow us to improve the situation of patients with this disease in the future.

**INSURANCE**

The Promoter of the clinical trial has an insurance policy that complies with current legislation and that will provide compensation in case of impairment of their health or injuries that may occur in relation to their participation in the clinical trial.

For more information regarding this section, please consult the principal investigator of the study at your site.

We inform you that it is possible that your participation in this clinical trial will modify the general and particular conditions (coverage) of your insurance policies (life, health, accident...). Therefore, we recommend that you contact your insurer to determine if participation in this study will affect your current insurance policy.

**PERSONAL DATA PROTECTION**

The treatment, communication and transfer of personal data of all participating patients will comply with the provisions of Regulation (EU) 2016/679 of the European Parliament and of the Council of April 27, 2016 on Data Protection (GDPR).

- Both the Center and the Promoter are respectively responsible for the processing of your data and undertake to comply with the data protection regulations in force. The Research Ethics Committees, the representatives of the Health Authority in inspection matters and the personnel authorized by the Sponsor, may only access to verify personal data, clinical study procedures and compliance with the rules of good clinical practice (always keeping safe the confidential information).
- The Researcher and the Sponsor are obliged to keep the data collected for the study for at least 25 years after its completion. Subsequently, your personal information will only be kept by your healthcare center and by the sponsor for other scientific research purposes if you have given your consent to do so, and if so permitted by law and applicable ethical requirements.
- In accordance with the provisions of the aforementioned legislation, you can exercise the rights of access, rectification, opposition and deletion of your data, for which you should contact your study doctor. In addition to the rights that you already know (access, modification, opposition and cancellation of data), you can now also limit the processing of data that are incorrect, request a copy or transfer to a third party (portability) the data that you have provided to the study. To exercise your rights, contact the main researcher of the study, the Dr. or the Data Protection Officer of the center: _______________________________________________________________________. We remind you that the data cannot be deleted, even if you stop participating in the trial, to ensure the validity of the research and comply with legal duties and drug authorization requirements. You also have the right to contact the Data Protection Agency if you are not satisfied.
- The data collected for the study will be identified by a code, so that information that can identify you is not included, and only your study doctor / collaborators will be able to relate data to you and to your medical history. Therefore, your identity will not be revealed to any other person except to the health authorities, when required or in cases of medical emergency. The Research Ethics Committees, the representatives of the Health Authority in matters of inspection and the personnel authorized by the Sponsor, may only access to verify personal data, clinical study procedures and compliance with the rules of good clinical practice (always keeping safe the confidential information).
- If we transfer your encrypted data outside the EU to the entities of our group, service providers or scientific researchers who collaborate with us, the participant's data will be protected with safeguards such as contracts or other mechanisms by the protection authorities.

**EXPENSES AND FINANCIAL COMPENSATION**

You will not have to pay for the therapy to be administered or for specific study tests. Your participation in the study will not entail any additional cost to the usual clinical practice.

**OTHER RELEVANT INFORMATION**

Any new information regarding the treatment that we are going to use in the clinical trial, and that may affect your participation, will be communicated to you as soon as possible.

If you decide to withdraw your consent to participate in this clinical trial, no new data will be added to the database, and you may require the destruction of all stored samples to prevent further testing.

You should also know that you can be excluded from the clinical trial if the sponsor or the researchers consider it appropriate for safety reasons, for any adverse event that occurs due to the treatment or because they consider that the established procedures are not being followed. In either case, you will be explained why you are leaving the study. By signing this consent form, you agree to comply with the clinical trial procedures that have been explained to you.

This consent document and the study have been approved by a Drug Research Ethics Committee.

If you have any problems or questions to ask about the clinical trial, your rights as a participant, or about any injury related to the clinical trial, you should contact:

**Dr. _______________________________**

**__________________ Hospital Service _______________________________**

**Telephone: ____________________________**

**INFORMED CONSENT FOR PATIENT OVER 18 YEARS OLD**

Phase I / II, multicenter, dose escalation clinical trial to evaluate the safety of an infusion of Natural Killer cells or memory T cells as adoptive therapy in coronavirus pneumonia and / or lymphopenia.

Phase II. Safety and effectiveness phase

Me,................................................ ................................................. (Name and surname)

- I have read the information sheet that has been given to me.
- I have been able to ask questions about the clinical trial.
- I have received satisfactory answers to my questions.
- I have received enough information about the clinical trial.

I understand that my participation is voluntary.

I understand that I can withdraw from the clinical trial:

1st Whenever you want.

2nd Without having to explain.

3rd Without this affecting your medical care.

I want the study doctor to communicate to me the information derived from the research that may be relevant and applicable to my health or that of my relatives, at the end of the study:

⃝ YES ⃝ NO Contact phone or email: ___________________________________

I consent to the storage and use of biological samples and associated data for future research under the conditions explained in this information sheet:

⃝ YES ⃝ NO Contact phone or email: ___________________________________

I consent to be contacted in the case of needing more information or additional biological samples:

⃝ YES ⃝ NO Contact phone or email: ___________________________________

I will receive a signed and dated copy of this informed consent document. I agree to participate in this clinical trial and I give my consent for access to and use of the data under the conditions detailed in this document.

Patient's signature

Signature Date

Signature of the responsible doctor

Signature Date

In _________________ to __________of_________________of 20__

This document will be signed in duplicate, keeping one copy for the researcher and another for the patient.
