# Supplementary material for: A phase I/II dose-escalation multi-center study to evaluate the safety of infusion of natural killer cells or memory T cells as adoptive therapy in coronavirus pneumonia and/or lymphopenia: RELEASE study protocol
Source: Trials. 2021 Oct 2;22:674. doi: 10.1186/s13063-021-05625-7 (PMC8487326; doi:10.1186/s13063-021-05625-7)
Supplement: Supplementary file 3 — Additional file 3. Supplementary Tables 1 and 2. [file 13063_2021_5625_MOESM3_ESM.docx]

**Supplementary table 1: WHO Trial Registration Data Set.**

| **1. Primary Registry and Trial Identifying Number** | ClinicalTrials.gov  Registry number: NCT04578210 |
| --- | --- |
| **2. Date of Registration in Primary Registry** | October 8, 2020 |
| **3. Secondary Identifying Numbers** | RELEASE |
| **4. Source(s) of Monetary or Material Support** | Fundación Cris Contra El Cáncer |
| **5. Primary Sponsor** | Dr Antonio Pérez Martínez |
| **6. Secondary Sponsor(s)** | No secondary sponsors in this study |
| **7. Contact for Public Queries** | Víctor Alelú Hernández, Mr  Email: [victor.alelu.ucicec@gmail.com](mailto:victor.alelu.ucicec%40gmail.com?subject=NCT04578210,%20RELEASE,%20Safety%20Infusion%20of%20NatuRal%20KillEr%20celLs%20or%20MEmory%20T%20Cells%20as%20Adoptive%20Therapy%20in%20COVID-19%20pnEumonia%20or%20Lymphopenia)  Phone: 91 497 53 59  Hospital Universitario La Paz  Madrid, Spain, 28046 |
| **8. Contact for Scientific Queries** | Antonio Pérez Martínez, Dr  (Principal Investigator)  Email: [aperezmartinez@salud.madrid.org](mailto:aperezmartinez@salud.madrid.org)  Phone: +34917277223;  Hospital Universitario La Paz  Madrid, Spain, 28046  Affiliation: Paediatric Haemato-oncology Department, University Hospital La Paz, Madrid, Spain. Hospital La Paz Institute for Health Research, IdiPAZ, University Hospital La Paz, Madrid, Spain. Faculty of Medicine Universidad Autónoma de Madrid, Madrid, Spain. |
| **9. Public Title** | Safety Infusion of NatuRal KillEr celLs or MEmory T Cells as Adoptive Therapy in COVID-19 pnEumonia or Lymphopenia |
| **10. Scientific Title** | A Phase I/II Dose-escalation Multi Center Study to Evaluate the Safety of Infusion of NatuRal KillEr celLs or MEmory T Cells as Adoptive Therapy in coronaviruS pnEumonia and/or Lymphopenia |
| **11. Countries of Recruitment** | Spain |
| **12. Health Condition(s) or Problem(s) Studied** | Corona Virus Infection |
| **13. Intervention(s)** | **Phase I: Not ramdomized Single Ascending Dose.**  The investigator assigns arm A or B to the patient taking into account the HLA data. If one or more HLA class I match between donor and recipient is present, patients will receive memory T cells - arm A-, in case they have less than one HLA class I match or HLA-KIR mismatch, patients will receive NK cells -arm B-.  In both arms, participants will subsequently undergo a correlative allocation into the different dose-escalating cohorts.  **Arm A:**   - Cohort 1: starting dose will be up to 1x10^5^/kg of memory T cells - Cohort 2: 1x10^5^/kg to 5x10^5^/kg of memory T cells - Cohort 3: 5x10^5^/kg to 1x10^6^/kg of memory T cells   **Arm B:**   - Cohort 1: starting dose will be up to 1x106/kg NK cells - Cohort 2: 1x106/kg to 5x106/kg NK cells - Cohort 3: 5x106/kg to 1x107/kg NK cells   Additionally to the investigational product, all patients will receive the local standard of care for COVID-19 treatment.  **Phase II: Ramdomized to Standard of Care Treatment (SoC) vs SoC + Recommended Phase 2 Dose (RP2D) (selected in the Phase I).**  The investigator assigns arm A or B to the patient taking into account the HLA data (same as in Phase I).  **Arm A:** Patients will be randomized to receive one of the following treatments:   - SoC - SoC + RP2D for memory T cells   **Arm B:** Patients will be randomized to receive one of the following treatments:   - SoC - SoC + RP2D for NK cells |
| **14. Key Inclusion and Exclusion Criteria** | **Inclusion criteria:**   - Male or female patients ≤ 80 years old. - Diagnosis of COVID-19 infection with laboratory confirmation by reverse-transcription PCR (RT-PCR) of SARS-CoV-2. - Onset of symptoms < 12 days prior to administration of study treatment. - **Phase I criteria:** Patients requiring hospitalization for COVID-19, with diagnosed pneumonia with chest radiograph or computed tomography imaging and/or lymphopenia (absolute lymphocyte counts below 1.2 x 109cells /L) AND O2Sat ≤ 94% on room air at screening, no oxygen requirement or with an oxygen need of ≤ 2.5 lpm in nasal cannula. - **Phase II criteria:** Patients requiring hospitalization with pneumonia diagnosed with chest radiograph or computed tomography imaging or lymphopenia (absolute lymphocyte counts below 1.2 x 109cells /L) AND O2Sat ≤ 94% on room air at screening, requiring or not oxygen supplementation (nasal cannula, oxygen mask with reservoir, non-invasive ventilation, etc), but excluding mechanical ventilation. - Have a negative pregnancy test documented prior to enrolment (for females of childbearing potential). - Be willing and able to comply with study procedures. - Patients must have the ability to comprehend and sign the informed consent. - Written informed consent obtained prior to any screening procedures.   **Exclusion criteria:**   - Enrolled in another Clinical Trial for COVID19. - Rapidly progressive disease with anticipated life-expectancy <72 hours. - Patients requiring mechanical ventilation. - Patients with multiorgan failure. - Moderate - severe (grade ≥ 3) organ impairment (liver, kidney), according to criteria from the National Cancer Institute (NCI CTCAE version 5.0). - Severe and/or uncontrolled concurrent medical disease that could cause unacceptable safety risks or compromise compliance with the protocol in the opinion of the clinical investigator. - Have a known history of human immunodeficiency virus infection, Hepatitis B or Hepatitis C; testing is not required in the absence of prior documentation or known history. - Pregnant or breastfeeding women, where pregnancy is defined as the state of a female after conception and until the termination of gestation, confirmed by a positive hCG laboratory test. - Any other condition that may interfere with the efficacy and/or safety evaluation of the trial according to the investigator’s opinion. |
| **15. Study Type** | Interventional, open label, multicenter study.  Phase I is an open label, not ramdomized, multicenter, dose escalation study in patients allocated in two different arms (arm A and B) taking into account the HLA class I match between donor and recipient .  Phase II is an open label, multicenter, double-arm, ramdomized study design, in patients allocated in two different arms (arm A and B) taking into account the HLA class I match between donor and recipient. |
| **16. Date of First Enrollment** | 4th September 2020 |
| **17. Sample Size** | 182 |
| **18. Recruitment Status** | Recruiting |
| **19. Primary Outcome(s)** | **Phase I:**  Occurrence of DLTs in all patients during the study treatment, until 21 days after cell infusion and the MTD [ Time Frame: 3 months ]  DLT: Any grade 3 or higher toxicity with an attribution of definitely or probably related to the infusion of the cells and any lower grade toxicity that increases to a grade 3 or higher as a direct result of the cell infusion.  Based on this, RP2D will be defined as: The recommended dose will be the MTD unless no MTD is determined in the dose escalation segment of the study. In the latter, the recommended dose will be the highest dose evaluated in the dose escalation segment.  **Phase II:**  The incidence of patient recovery infusing adoptive NK cells or adoptive memory T cells.  Recovery is defined as: Proportion of participants in each group with normalization of fever and oxygen saturation [criteria for normalization: temperature < 38°C armpit, and SpO2 > 94%, sustained for at least 24 hours] or lymphopenia recovery through Day 14. |
| **20. Key Secondary Outcomes** | - Time (days) to normal level of lymphocytes. - Proportion of patients showing clinical improvement at day 7 according to the investigator (based on respiratory status and blood-result test). - Proportion of patients receiving a second cycle. - Time (days) to first negative SARS-CoV-2 PCR after infusing adoptive NK cells or adoptive memory T cells. - The incidence of treatment-related adverse events (new or worsening from baseline) will be summarized by system organ class and/or preferred term, severity, type of adverse event and relation to study treatment. They will be defined by the Common Terminology Criteria for Adverse Events (CTCAE) Version 5.0 - Duration (days) of hospitalization. - Time (days) to discharge or to a NEWS of ≤ 2 and maintained for 24 hours, whichever comes first. - Time to improvement by one category on a 7-point ordinal scale. - Subject clinical status (on a 7-point ordinal scale) at day 14. - Proportion of patients requiring intensive |
| **21. Ethics Review** | The study protocol version 1.0 of the 1st of April 2020 was approved by the Ethics Committee CEIm Hospital Universitario La Paz (Identifier: Clinical Ethical Approval No. HULP-5579) on the 8th April 2020. |
| **22. Completion date** | April  2022 |
| **23. Summary Results** | Phase I results are published in Pérez-Martínez A, Mora-Rillo M, Ferreras C, Guerra-García P, Pascual-Miguel B, Mestre-Durán C, et al. Phase I dose-escalation single centre clinical trial to evaluate the safety of infusion of memory T cells as adoptive therapy in COVID-19 (RELEASE). EClinicalMedicine. 2021 Sep;39:101086. doi: 10.1016/j.eclinm.2021.101086. Epub 2021 Aug 13. PMID: 34405140; PMCID: PMC8361305. |
| **24. IPD sharing statement** | There is no plan to share IPD at present. |

SUPPLEMENTARY TABLE 2: Definition of outcomes

| Outcome | Metric | Estimator | Time point |
| --- | --- | --- | --- |
| PHASE I PRIMARY OUTCOMES: | | | |
| Occurrence of DLTs in all patients | Frequency | Proportion (%) and confidence interval | Until 21 days after cell infusion |
| Maximum tolerated dose (MTD) | Nºcells/kg | Nºcells/kg | After phase I is complete |
| PHASE I CO-PRIMARY OUTCOME: | | | |
| Incidence and nature of DLT of a single infusion of NK or memory T cells from a healthy donor recovered from COVID-19 | Frequency | Proportion of participants in each group with normalization of fever and oxygen saturation (criteria for normalization: temperature < 38°C armpit, and SpO2 > 94%, sustained for at least 24 hours) or lymphopenia recovery | Day 14 |
| PHASE II PRIMARY OUTCOMES: | | | |
| Incidence of patient recovery infusing adoptive NK cells or adoptive memory T cells | Frequency | Proportion (%) and confidence interval | Day 14 |
| PHASE 2 SECONDARY OUTCOMES: | | | |
| Additional biochemical information for the patient evolution | Time to normal level of lymphocytes, time to negative SARS-CoV-2 test… | Median (IQR*) and confidence interval |  |
| General status of the patient | Visits to intensive unit care, clinical status...). | Proportion (%), median and confidence interval |  |
| Exploratory objectives | | | |
| Related with immune reconstitution | Immunoglobulins, serum cytokines, T cells, NK cells and B cells repertoire | Median (IQR*) and confidence interval |  |
| Determination of donor chimerism |  | Proportion (%) and confidence interval |  |

*IQR = InterQuartile Range
